# Gene Expression Profiling and Network Analysis Reveals Lipid and Steroid Metabolism to Be the Most Favored by TNFα in HepG2 Cells

**DOI:** 10.1371/journal.pone.0009063

**Published:** 2010-02-04

**Authors:** Amit K. Pandey, Neha Munjal, Malabika Datta

**Affiliations:** Institute of Genomics and Integrative Biology (Council of Scientific and Industrial Research), Delhi, India; Karolinska Institutet, Sweden

## Abstract

**Background:**

The proinflammatory cytokine, TNFα, is a crucial mediator of the pathogenesis of several diseases, more so in cases involving the liver wherein it is critical in maintaining liver homeostasis since it is a major determiner of hepatocyte life and death. Gene expression profiling serves as an appropriate strategy to unravel the underlying signatures to envisage such varied responses and considering this, gene transcription profiling was examined in control and TNFα treated HepG2 cells.

**Methods and Findings:**

Microarray experiments between control and TNFα treated HepG2 cells indicated that TNFα could significantly alter the expression profiling of 140 genes; among those up-regulated, several GO (Gene Ontology) terms related to lipid and fat metabolism were significantly (p<0.01) overrepresented indicating a global preference of fat metabolism within the hepatocyte and those within the down-regulated dataset included genes involved in several aspects of the immune response like immunoglobulin receptor activity and IgE binding thereby indicating a compromise in the immune defense mechanism(s). Conserved transcription factor binding sites were identified in identically clustered genes within a common GO term and SREBP-1 and FOXJ2 depicted increased occupation of their respective binding elements in the presence of TNFα. The interacting network of “lipid metabolism, small molecule biochemistry” was derived to be significantly overrepresented that correlated well with the top canonical pathway of “biosynthesis of steroids”.

**Conclusions:**

TNFα alters the transcriptome profiling within HepG2 cells with an interesting catalog of genes being affected and those involved in lipid and steroid metabolism to be the most favored. This study represents a composite analysis of the effects of TNFα in HepG2 cells that encompasses the altered transcriptome profiling, the functional analysis of the up- and down- regulated genes and the identification of conserved transcription factor binding sites. These could possibly determine TNFα mediated alterations mainly the phenotypes of hepatic steatosis and fatty liver associated with several hepatic pathological states.

## Introduction

The multifunctional proinflamatory cytokine, TNFα (Tumor Necrosis Factor α) has effects on lipid metabolism, coagulation, insulin resistance, and endothelial function and therefore is important in cellular pathophysiology. It induces the activation of predominantly two signaling pathways within the cell – one involving the transcription factor NF-κB (Nuclear Factor-κB) that lies inactive in the cytosol and is activated on TNFα binding to its receptor on the membrane and the other involves the MAPK (Mitogen Activated Protein Kinase) pathway [1]–[3]. Both these pathways underlie the basic mechanism(s) of TNFα action in several pathological conditions. TNFα is widely implicated in various diseases. Although primarily known to be associated with inflammation, TNFα now occupies a central place in the crosstalk of many pathological states. It plays a major role in muscular abnormalities resulting in muscle wasting [4], idiopathic pulmonary fibrosis [5], rheumatoid arthritis [6], Crohn's disease [7] and psoriasis [8]. Several other diseases like cerebral malaria [9], asthma [10], [11] and cystic fibrosis [12] also have a strong correlation to TNFα levels. Chronic inflammation is also frequently associated with the metabolic syndrome and TNFα is one of the significant mediators between the two states [13]. The metabolic syndrome is a cluster of metabolic abnormalities leading to increased risk for cardiovascular diseases and diabetes type 2 and herein visceral obesity and the resulting insulin resistance are the major determinants in the development of this syndrome [14].

The liver is central to metabolic control and actively participates in storing and releasing glucose, nitrogen metabolites and lipids as and when required. It is also majorly involved in detoxification mechanisms and is, therefore, no doubt significant for proper functioning of the body and any liver injury is extremely detrimental in preserving normal hepatic homeostasis. Liver injury and death are the major hallmarks in all liver diseases [15] and it is during these conditions that the liver is exposed to increased levels of TNFα apart from other inflammatory cytokines. Serum and hepatic TNFα levels are increased in patients with HBV and HCV infection [16]–[18] and also in subjects with detectable liver failure and other liver injuries [19], [20]. In all these cases, TNFα along with other cytokines participate in initiating a wound healing response [15] or mediate hepatocyte damage [21], [22]. Paradoxically, although harmful to the injured hepatocyte, TNFα induces healthy hepatocytes to proliferate rather than die [23]. This especially occurs as a conditioned response against any hepatectomy or liver regeneration. Thus to maintain normal liver integrity and homeostasis by either hepatocyte death as observed during liver injury or by inducing hepatocyte proliferation during liver regeneration depends on whether TNFα-induced apoptosis or cell survival pathways, respectively are predominant. All these imply that TNFα is very critical in liver function and its increased levels exert several pleiotropic effects thereby emphasizing the need for a detailed understanding of hepatic TNFα action [15].

In view of these varied effects of TNFα, we sought to study the TNFα-induced alteration in gene expression patterns within the hepatocyte, the common pathways they fall in and to find a correlation among co-regulated genes. Our results demonstrate a significant number of hepatic genes to be statistically altered by TNFα and the most significant pathway and network to be targeted predominantly involved those of lipid metabolism, cholesterol and steroid biosynthesis. Interestingly, conserved transcription factors' binding sites were found in these sets of genes that could determine the correlation between the altered gene set and the hepatic processes favored by TNFα. Although earlier studies [24], [25] have shown some specific effects of TNFα on HepG2 cells, our results demonstrate a wholesome analysis of TNFα effect on HepG2 cells that correlates the altered transcriptome profile with identification of conserved transcription factor binding sites that could determine several hepatic pathological states.

## Methods

### Cell Culture and Treatment

Experiments were done in HepG2 (human hepatocellular carcinoma) cells obtained from the National Centre for Cell Science, Pune, India. HepG2 cells retain several functions of the normal human liver including synthesis of albumin, lipoprotein and several other liver specific functions. Among these functions notable are those related to cholesterol and triglyceride metabolism. In several hepatic physiological states, inflammation within the liver is a significant accompanying phenomenon along with the accumulation of lipid vesicles. Dysregulation of liver metabolism and hepatic inflammation run in parallel in several liver diseases and thereby inflammation within the liver predisposes it to the onset of a number of hepatic pathologies. HepG2 cells are recognized as an *in vitro* human model system and are identified as a possible bioartificial liver [26] thereby corroborating their use as a resource to study several hepatic functions and for metabolic studies [27]. They were maintained in DMEM supplemented with 10% fetal calf serum with 1% antibiotic-antimycotic (100 units/ml penicillin, 0.1 mg/ml streptomycin and 0.25 µg/ml amphoterecin B) at 37°C in a humified atmosphere of 5% CO_2_. On attaining confluence, cells were serum starved overnight and incubated in the absence (control) and presence of TNFα (Sigma Chemical Co., St. Louis, USA; 0.5 nM, 12 h). The dose and time were chosen becausein HepG2 cells, we had observed optimum inhibition of insulin-stimulated Akt activation that plateaued thereafter at further dose and time points. The total levels of the Akt protein, however, remained constant (Figure S1).

### RNA Isolation

On termination of incubation, total RNA was isolated from cells using Trizol (Invitrogen) according to the manufacturer's instructions and subjected to a cleanup using RNeasy protect Mini Kit (Qiagen, Germany). RNA was quantified in a NANODROP spectrophotometer (ND 1000) and the ratios of OD260/280 nm were between 1.8–2.0. The integrity of RNA samples was determined in the Bioanalyser 2100 System (Agilent Technologies, Palo Alto, CA) and all samples had RIN (RNA integrity number) values between 8.0–8.5.

### Microarray and Data Analysis

Microarray experiments were performed in triplicate (three control and three TNFα- treated) at The Centre for Genomic Applications (TCGA), New Delhi, India according to the manufacturer's instructions (Affymetrix, Santa Clara, USA). Briefly, RNA (control and TNFα treated) was reverse transcribed to cDNA that was subjected to in vitro transcription to produce biotinylated cRNA that was hybridized to the human array chip (Human Genome U133 Plus 2.0, Affymetrix). Images were scanned using the GeneChip 3000 7G scanner (Affymetrix). Signal intensities and absolute call dataset were generated with Affymetrix Gene Chip Operating Software (GCOS) using the MAS5.0 algorithm. This resulted in normalized values of each chip to a target intensity of 500 as detailed in the Affymetrix statistical algorithm. The efficiency of the labeling reaction and hybridization performance was assessed by the optimal 3′/5′ hybridization ratios for the housekeeping genes, poly-A-spike in controls and the prokaryotic controls. Detection calls identified transcripts to be either reliably detected (Present) or below the background levels (Absent) if the detection p-value was above or below 0.05 respectively. Genes with present calls in at least 2 out of the three replicates were included in the analysis that was done with Microsoft Excel 2007 using the steps as described in http://jura.wi.mit.edu/bio/education/bioinfo2007/arrays/. The data were normalized such that the average expression of the present genes in all the samples was identical, log transformed and statistical analysis was done with student's *t-*test. Genes were filtered on the basis of call, fold change and p value. All genes with a signal log ratio >±0.5 (1.41 fold) and p value <0.05 were chosen to be statistically altered by TNFα. The 1.41-fold criterion as a minimum cutoff for change is chosen based on the literature reported earlier in HepG2 cells [28]. The study was in accordance to the MIAMI guidelines and the microarray data have been submitted to the Gene Expression Omnibus (GEO) database [29]. Accession number for the data is GSE12161.

### Real Time PCR

Real Time quantitative PCR was done for validation of gene expression microarray data. Total RNA (Control and TNFα-treated) was isolated as described above and 2 µg RNA from each set was reverse transcribed using MMuLV reverse transcriptase and random hexamers. 2 µl of this was amplified in a final volume of 25 µl using the SYBR Green Real Time PCR Master Mix (Applied Biosystems) and gene specific primers. After an initial activation at 50°C for 2 min and a preincubation step at 95°C for 10 min, reaction mixtures were subjected to 40 cycles of denaturation at 95°C for 15 sec, annealing at 56°C for 30 sec and extension at 72°C for 35 sec (ABI 7500). This was followed by a dissociation curve analysis at 95°C for 15 sec, 60°C for 20 sec and 95°C for 15 sec. Data analysis was done as described by Pfaffl, 2001 [30] and 18S rRNA was taken as the internal control. No RT control (without the reverse transcriptase) and a no template control (without the RNA template) were taken as the negative controls for the experiments. All experiments were done in triplicate.

### Gene Ontology (GO) Classification and Cluster Analysis

Genes that were found to be significantly altered by TNFα were classified into categories of biological processes and molecular functions using Gene Ontology Tree Machine (GOTM, [31]) and DAVID [32]. Genes over-represented in our dataset in the context of the reference set (Human Genome U133 Plus 2.0 chip, Affymetrix) were determined with the GO Tool Box [33] using Bonferroni correction. Genes were then clustered on the basis of their normalized expression intensities using the *Avadis* software (version 3.1, Stratagene).

### Promoter and Pathway Analysis

To identify the likely identical regulatory controls among the altered genes, genes common to a single cluster and Gene Ontology term were taken for promoter analysis. 5.0 kb upstream regions from the transcription start sites of such genes were retrieved using Ensembl [34] and the transcription factor(s) binding within this region was determined using Over-represented Transcription Factor Binding Site Prediction (OTFBS, Release 6.0.) tool [35] that detects over-represented motifs of known transcription factors within groups of related sequences. This was done considering the fact that genes with similar function as well as expression profiles are more likely to be under the same regulatory control than genes within either of the categories [36]. Also to categorically determine which specific pathways the altered genes map to and to determine the interacting network among them, if any, the dataset containing the gene identifiers alongwith the corresponding fold changes were uploaded into the Ingenuity Pathways Analysis program. Each identifier was mapped to its corresponding gene object in the Ingenuity knowledge base. A 1.41 fold change (log ratio ±0.5) was set to identify genes whose expression was significantly differentially regulated. These genes, called focus genes, were overlaid onto a global molecular network developed from information contained in the Ingenuity knowledge base. Networks of these focus genes were then algorithmically generated based on their connectivity. A graphical representation of the molecular relationships between these genes is shown where genes or gene products are represented as nodes, and the biological relationship between two nodes is represented as an edge (line). All edges are supported by at least 1 reference from the literature, from a textbook, or from canonical information stored in the Ingenuity knowledge base. Human, mouse, and rat orthologs of a gene are stored as separate objects in the Ingenuity knowledge base, but are represented as a single node in the network. The intensity of the node color indicates the degree of up- (red) or down- (green) regulation. Canonical Pathways Analysis identified the pathways from the Ingenuity Pathways Analysis library of canonical pathways that were most significant to the dataset. Genes from the dataset with a 1.41 fold change (log_2_ ratio ±0.5) were associated with a canonical pathway in the Ingenuity knowledge base and considered for the analysis. The significance of the association between the dataset and the canonical pathway was measured in 2 ways: 1) A ratio of the number of genes from the dataset that met the expression value cutoff that map to the pathway divided by the total number of molecules that exist in the canonical pathway is displayed. 2) Fischer's exact test was used to calculate a p-value determining the probability that the association between the genes in the dataset and the canonical pathway is explained by chance alone.

### Electrophoretic Mobility Shift Assay (EMSA)

The electrophoretic mobility shift assay analysis was done essentially as described by Shiraga et al. [37]. Briefly nuclear extracts were prepared from control and TNFα-treated (0.5 nM, 12 h) HepG2 cells by lysing the cell pellets in a buffer containing 10 mM HEPES (pH 7.4), 1.5 mM MgCl_2_, 10 mM KCl, 1 mM DTT, 0.1 mM EDTA, 0.1 mM EGTA, 0.25% Nonidet P-40 and protease inhibitor cocktail (final concentration 1X, Sigma, St. Louis, MO, USA) and incubating in ice for 15 min. These were then centrifuged at 10,000 g for 15 min at 4°C. The pellets obtained were resuspended in nuclear lysis buffer (20 mM HEPES (pH 7.4), 1.5 mM MgCl_2_, 40 mM NaCl, 1 mM DTT, 0.1 mM EDTA, 0.1 mM EGTA, 25% glycerol and protease inhibitor cocktail (final concentration 1X, Sigma, St. Louis, MO, USA)) and incubated on ice for 30 min with intermittent shaking. The supernatant obtained after centrifugation of the nuclear lysates at 17,000 g for 15 min at 4°C were taken as the nuclear fraction and used for the binding studies.

Double stranded DNA was generated by mixing equimolar amounts of the complementary oligonucleotides in annealing buffer (Ambion, CA, USA). Mutated sequences of the transcription factors were also used to check the specificity of the binding of the transcription factors. These annealed double stranded DNA were labeled with [γ^32^-P]-ATP (BRIT, Hyderabad, India) in the presence of T4-polynucleotidekinase (New England Biolabs, MA, USA) according manufacturer's instructions. Nuclear extracts (15 µg) from the control and TNFα-treated cells were incubated for 30 min at room temperature in the presence of reaction buffer containing 8 mM Tris-KCl (pH 8.0), 2 mM EDTA, 1 mM DTT, 12% glycerol, 1 µg BSA and 1 µg of poly(dI-dC). Either the wild type or mutated labeled double stranded oligonucleotide (40,000 cpm) was then added to the reaction mixture and incubated for 45 min at room temperature. On termination of incubation, samples were loaded onto a non-reducing 5% polyacrylamide gel and electrophoresed in 0.5XTBE. Gels were then dried and subjected to phosphorimager analyses (FLA 2000, Fujifilm, Japan). The densitometric analyses were done using the Alpha DigiDoc 1201 software (Alpha Innotech Corporation, CA, USA). The same size rectangle box was drawn surrounding each band and the intensity of each was analyzed by the program after subtraction of the background intensity.

### Cholesterol Synthesis

On attaining confluence, HepG2 cells were incubated in the absence or presence of TNFα (0.5 nM, 12 h). Additionally to determine which specific step of the steroid biosynthetic pathway is the most significant contributor for TNFα action herein, gene specific siRNAs were used to categorically knock down specific genes. HepG2 cells were transfected with 50 nM of either control or specific siRNAs (Santa Cruz, CA, USA) according to the manufacturer's instructions. After allowing the cells to grow in fresh DMEM for 48 h, they were incubated with TNFα (0.5 nM, 12 h) and on termination of incubation, cells were lysed and lipids were extracted by addition of 1.0 ml of chloroform: methanol (2∶1). These lipid extracts were vacuum dried and the residues were dissolved in 2-propanol containing 10% Triton-X-100. Cholesterol was estimated in an analyser (COBAS INTEGRA 400 plus, Roche, Germany) according to the manufacturer's instructions. Results are expressed after normalization to the total protein content (DC Protein Assay Kit, BIORAD, CA).

### Data Analysis

Microarray data from three replicate experiments generated by GCOS were exported to Microsoft Excel 2007 for further analysis and is described above. A value of p<0.05 was taken to be statistically significant. Only those genes with present calls in at least 2 out of 3 replicates were analyzed and a cutoff value for differential gene expression was defined as transcripts that showed a>1.41 fold change and a t-test p value of <0.05. RT-PCR was done in triplicates and data presented are means± S.E.M of three independent experiments. Statistical significance between the control and treated groups was determined using Student's t-test where p≤0.05 was considered as statistically significant.

## Results

### TNFα Induced Alteration in Gene Expression in HepG2 Cells

**Figure 1:**
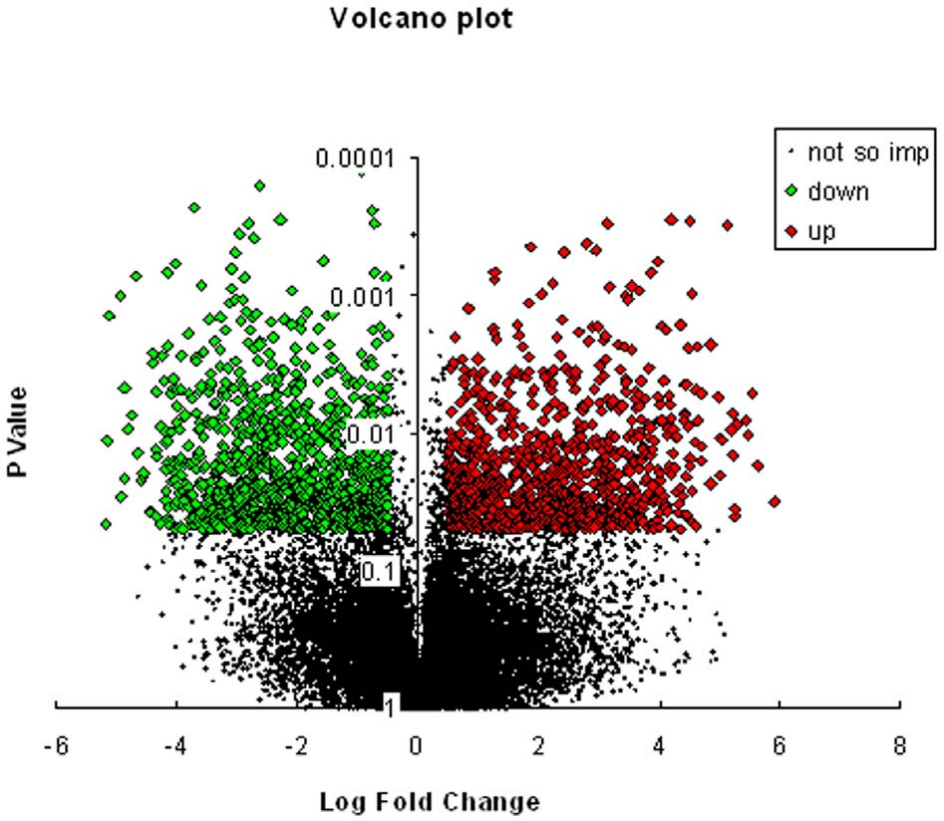
A volcano plot of genes altered by TNFα in HepG2 cells. Log_2_ fold changes and their corresponding p-values of all genes in the microarray were taken for construction of the volcano plot. Genes up-regulated with more than 1.5 fold change with a p-value <0.05 are depicted in red boxes and those down regulated with identical fold change and p-value are in green boxes. All other genes in the array that were not found to be significantly altered are in black dots.

To identify genes altered by TNFα in HepG2 cells, mRNA expression in these cells incubated in the absence and presence of TNFα (0.5 nM, 12 h) was analysed using whole genome oligonucleotide expression arrays (Human Genome U133 Plus 2.0, Affymetrix). TNFα treatment significantly (p<0.05) altered the expression of 140 genes in HepG2 cells. Figure 1 shows the volcano plot of the microarray hybridization with the significantly up-regulated genes being represented with red colored boxes and those down regulated with green ones. Those that were not significantly altered are depicted in black dots. These altered genes were classified into functional categories of biological processes using Gene Ontology Tree Machine (GOTM) and DAVID. The GO database has a hierarchical nature such that genes annotated with a specific node (GO term) are also annotated with every ancestor of that node with the result that the genes map onto more than one GO term. A functional GO class thereby included genes assigned in the listed GO term by the GO consortium or a more specific break-up of the listed term that reflects in the gene being classified in more than one GO term. From our dataset, genes that clearly mapped onto a single GO term were assigned as such and for others that mapped onto more than one GO term, we put genes in the term where there was experimental validation available and when not, we put them in a particular GO class considering their functional closeness to other members of that class. Table S1 gives the Gene Ontology terms, the fold change and the p value of the significantly altered genes. Of these, 67 genes were up-regulated and 73 were down-regulated with a minimal fold change of ±1.41 (log_2_ ratio ≥±0.5). Genes involved in cell adhesion like Laminin (gamma 1, −1.4 fold), Tao kinase2 (−1.6 fold) and Fibroblast Growth Factor Receptor-Like 1 (−1.5 fold) were significantly down regulated by TNFα. Almost all the genes involved in response to stimulus (HSPA1B, ORM2, MST1, H3/O and C4B) and regulation of biological processes (PUM1, CEBPα and BRPF1) were down regulated. While some genes of signal transduction like FCER1G, RASSF7, NR1H4 and RGL3 were significantly (p<0.05) down regulated, others of the same category like MKNK2, RYK, NFKB2, IL22RA1, THRAP6 and KISS1R were upregulated. Further upregulated genes were involved in transport such as GOSR2 (+1.4 fold), HCN3 (+1.7 fold), SYTL1 (+1.4 fold), SLC43A2 (+1.9 fold) and SLC27A5 (+2 fold) and lipid and carbohydrate metabolism like FDPS (+1.6 fold), PAFAH2 (+2 fold), FADS (+1.7 fold), SQLE (+2.5 fold), HSD17B7 (+1.7 fold) and IDH2 (+1.5 fold). While genes involved in carbohydrate metabolism, for e.g. AKR1B1, GNPDA2, and PDK3 were significantly downregulated, some genes of nucleic acid and amino acid metabolism like PAH, NDUFS1, DHFR and TNRC9 were upregulated. Others like STOM, UBE2J1, SCRN3 (protein metabolism), SIRT6, ELF5, DNMT3B (nucleic acid metabolism), AP3B1, SLC6A12, STXBP5, AP1S2, AQP3 (transport) and TIMP1 were significantly downregulated. Interestingly genes involved in cellular homeostasis i.e. calreticulin (−1.6 fold) and protein disulfide isomerase (−1.9 fold) were also downregulated.

### Real Time PCR Validation of Microarray Data

**Figure 2:**
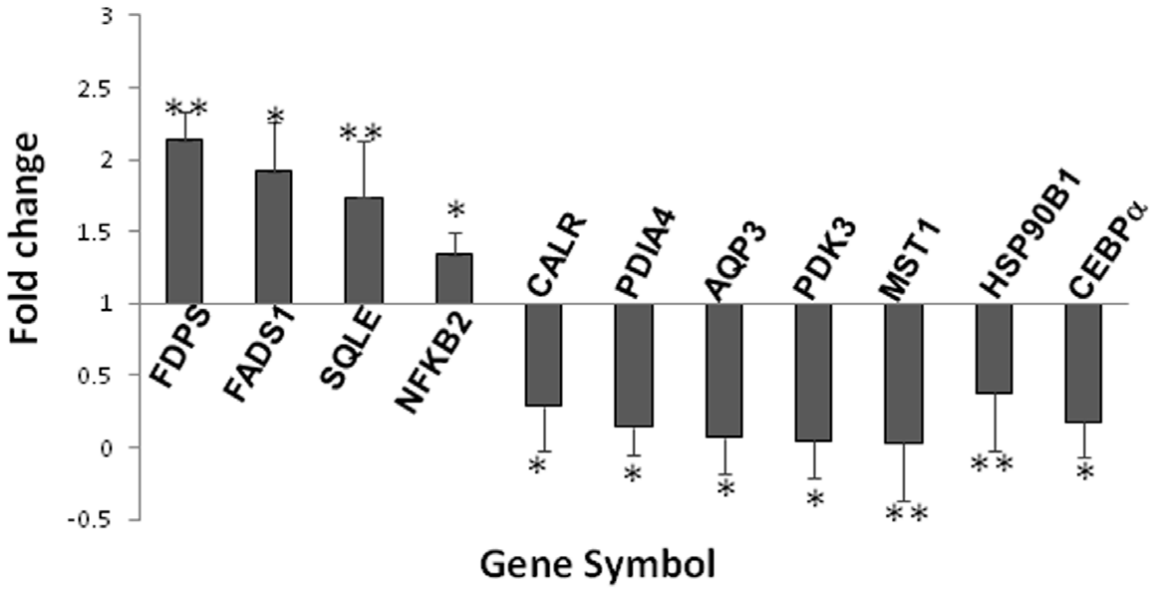
Validation of microarray gene expression data by Real Time PCR. HepG2 cells were treated in the absence and presence of TNFα (0.5 nM, 12 h) and the isolated total RNA was subjected to Real time PCR with gene specific primers as listed in Table 2. Values of each gene were normalized to those of 18S rRNA and are plotted with respect to the control that has arbitrarily been taken as 1. Each value is the mean ± SEM of three independent experiments. *p<0.01 as compared to control. **p<0.05 as compared to control.

Quantitative real time PCR was done to validate a group of selected up- and down-regulated genes. The primer sets used are shown in Table 1. The experiment was done for FADS1, SQLE, PDIA4, HSP90B, CEBPα, CALR, FDPS, MST1, PDK3, AQP3 and NFKB2. As in the results of the microarray analysis, all these genes depicted an identical pattern of alteration as compared to the control. In TNFα treated cells, PDIA4, HSP90B1, CEBPα, CALR, MST1, PDK3 and AQP3 showed an inhibition of the transcript levels while FADS1, SQLE, FDPS and NFKB2 showed increased levels (Figure 2).

**Table 1:** Primers used for microarray gene expression validation.

Gene Symbol	Forward primer 5′-3′	Reverse primer 5′-3′	AmpliconSize(bp)
HSP90B1	AATCTGGGACAAGCGAGTTTTT	GCGGAATAGAAACCGACACCA	103
FDPS	CCAAGAAAAGCAGGATTTCGTTC	CCGGGCAATAGCATCTCCTA	100
PDIA4	TCCCATTCCTGTTGCCAAGAT	GCCCTCGTAGTCTACAGCCT	121
SQLE	CCACTGACTGTTGTTGCAGAT	TGAGAACTGGACTCGGGTTAG	253
FADS1	TTGTACCTCCATTGGCTTCC	CAGCCTATGCCCAAAGCTAC	122
CALR	GTTTCGAGCCTTTCAGCAAC	TCTGAGTCTCCGTGCATGTC	142
CEBPα	ACGATCAGTCCATCCCAGAG	TTCACATTGCACAAGGCACT	122
PDK3	TGGTTCCTACAATGGCACAA	GGAAAGAGATGCGGTTGGTA	120
AQP3	GAGCATCCACTGACTGTCCA	AAATCCTGAAGGGGCTGTCT	116
MST1	CACAGCCAATACCACCACTG	GGTCTTTGCACGCGTATTTT	101
NFKB2	ACTACCTCCCACCTCGTCCT	ACGCCTCTTGACCTCACT	124

### Functional Classification of Genes Altered by TNFα in HepG2 Cells

**Figure 3:**
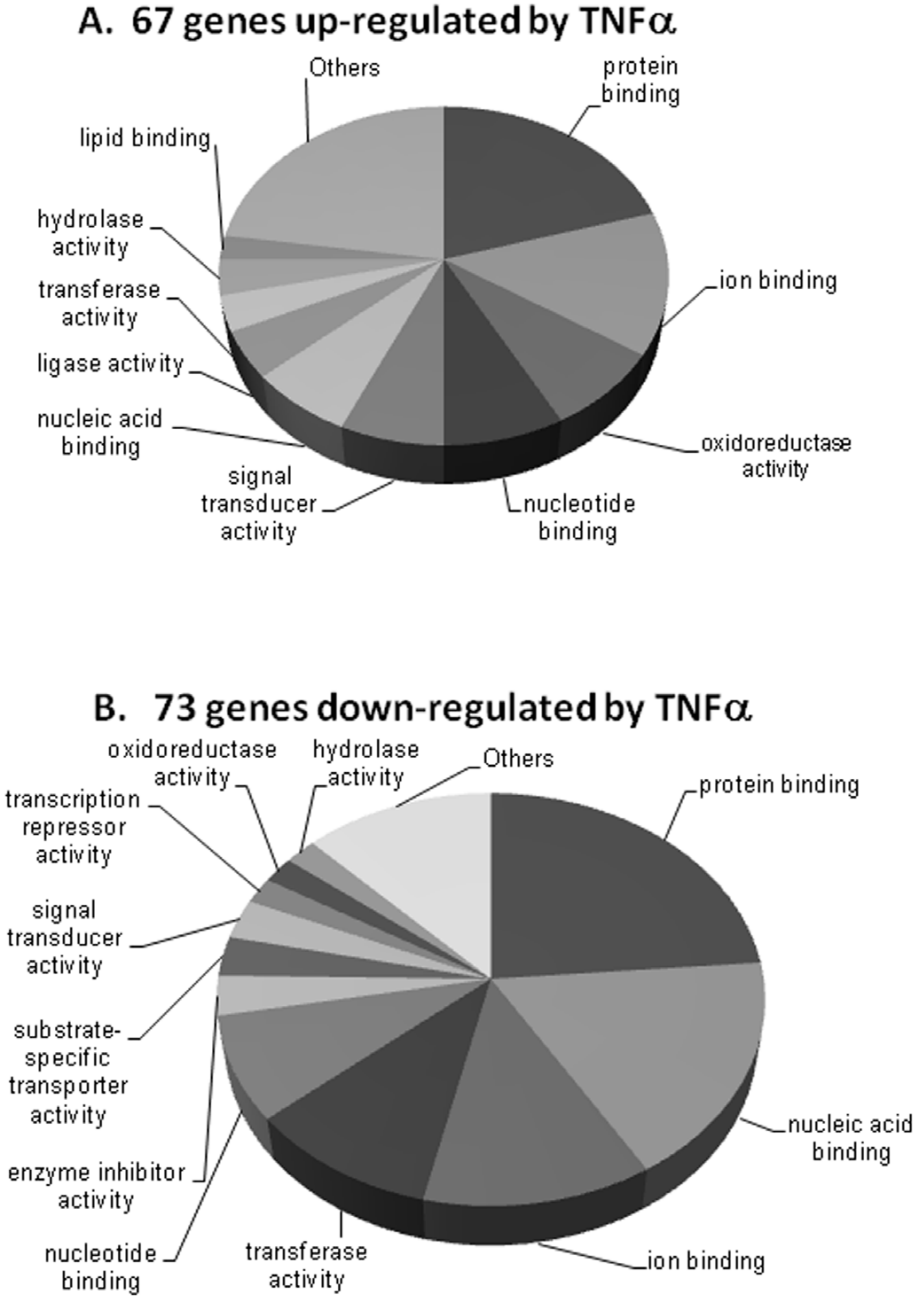
Classification of TNFα-regulated genes into functional groups. HepG2 genes that were altered by TNFα were classified on the basis of molecular functions using the GO Tool Box. Fraction of genes and the corresponding GO terms from the up-regulated set are shown in A and the same from the down regulated set are depicted in B.

All the 67 up-regulated and 73 down-regulated genes were separately grouped into molecular functions using the GO Tool Box. At Level 3, molecular functions that emerged specifically as being altered among the up-regulated genes were those of lipid binding and ligase activity while those of transcription repressor and enzyme inhibitor activities were specific to the down-regulated genes. Figures 3A and B depict respectively the fraction of TNFα induced up- and down- regulated genes in HepG2 cells among the various molecular functions as identified by the GO Tool Box. Significantly (p<0.01) over represented Gene Ontology terms (molecular function) in down- and up- regulated genes are shown in Tables 2A and B respectively. The tables give the number of genes with that gene ontology term on the Human Genome U133 Plus 2.0 array, the number of genes in the same gene ontology term in our altered dataset and the p value of the occurrence of that number of genes in the dataset. For genes up-regulated by TNFα, several gene ontology terms related to lipid and fat metabolism like keto-steroid reductase activity, cholate-CoA ligase activity, squalene monooxygenase activity, oxidoreductase activity were significantly (p<0.01) over represented indicating an overall favor of genes involved in fat metabolism within the hepatocyte by TNFα. GO terms involved with immune responses like IgE receptor activity, immunoglobulin receptor activity and IgE binding were also highly over represented in the down-regulated set indicating a compromise in immune defense mechanism(s). An identical analysis using the GO class: biological processes identified several genes belonging to the steroid and lipid metabolic processes to be significantly (p<0.01) over-represented among the up-regulated set and those very specifically involved in diverse aspects of the overall immune response to be significantly (p<0.01) over represented in the down-regulated set of genes (Table S2).

**Table 2:** Gene Ontology (Molecular Function) terms overrepresented (p<0.01) in the set of genes up- and down- regulated by TNFα.

**A. Overrepresentation of down-regulated genes**				
**GO_ID**	**TERM**	**NB_IN_REF**	**NB_IN_SET**	**pVAL**
GO:0003677	DNA binding	3525	14	0.000379
GO:0005515	protein binding	8667	23	0.001096
GO:0016429	tRNA (adenine-N1-)-methyltransferase activity	1	1	0.001522
GO:0019767	IgE receptor activity	1	1	0.001522
GO:0004361	glutaryl-CoA dehydrogenase activity	1	1	0.001522
GO:0008326	site-specific DNA-methyltransferase (cytosine-specific) activity	1	1	0.001522
GO:0016426	tRNA (adenine)-methyltransferase activity	1	1	0.001522
GO:0019763	immunoglobulin receptor activity	2	1	0.00304
GO:0004105	choline-phosphate cytidylyltransferase activity	2	1	0.00304
GO:0051082	unfolded protein binding	202	3	0.003357
GO:0004032	aldehyde reductase activity	3	1	0.004553
GO:0004740	(pyruvate dehydrogenase (lipoamide)) kinase activity	4	1	0.006061
GO:0030414	protease inhibitor activity	270	3	0.0073
GO:0008757	S-adenosylmethionine-dependent methyltransferase activity	87	2	0.007487
GO:0008168	methyltransferase activity	273	3	0.007515
GO:0019863	IgE binding	5	1	0.007565
GO:0016741	transferase activity, transferring one-carbon groups	277	3	0.007806
GO:0009008	DNA-methyltransferase activity	6	1	0.009065
GO:0005007	fibroblast growth factor receptor activity	6	1	0.009065
**B. Overrepresentation of up-regulated genes**				
**GO_ID**	**TERM**	**NB_IN_REF**	**NB_IN_SET**	**pVAL**
GO:0016616	oxidoreductase activity, acting on the CH-OH group of	160	3	0.001262
	donors, NAD or NADP as acceptor			
GO:0016919	nardilysin activity	1	1	0.001353
GO:0050576	3-keto-steroid reductase activity	1	1	0.001353
GO:0004506	squalene monooxygenase activity	1	1	0.001353
GO:0048408	epidermal growth factor binding	1	1	0.001353
GO:0047747	cholate-CoA ligase activity	1	1	0.001353
GO:0016614	oxidoreductase activity, acting on CH-OH group of donors	174	3	0.001597
GO:0004161	dimethylallyltranstransferase activity	2	1	0.002702
GO:0004337	geranyltranstransferase activity	2	1	0.002702
GO:0000247	C-8 sterol isomerase activity	2	1	0.002702
GO:0016491	oxidoreductase activity	1510	7	0.002977
GO:0004769	steroid delta-isomerase activity	3	1	0.004048
GO:0017147	Wnt-protein binding	3	1	0.004048
GO:0004505	phenylalanine 4-monooxygenase activity	3	1	0.004048
GO:0004090	carbonyl reductase (NADPH) activity	3	1	0.004048
**GO_ID**	**TERM**	**NB_IN_REF**	**NB_IN_SET**	**pVAL**
GO:0004146	dihydrofolate reductase activity	3	1	0.004048
GO:0004450	isocitrate dehydrogenase (NADP+) activity	4	1	0.00539
GO:0003847	1-alkyl-2-acetylglycerophosphocholine esterase activity	6	1	0.008064
GO:0016874	ligase activity	630	4	0.008635
GO:0042043	neurexin binding	7	1	0.009396

### Clustering and Promoter Analyses

TNFα induced up- and down- regulated genes were clustered hierarchically on the basis of their normalized expression intensities (Figure S2) and genes with the same GO functional term and within the same cluster were selected for further promoter analysis. Example results of genes from three GO terms i.e. “Metabolism”, “Signal Transduction” and “Regulation of Biological Process” that clustered together were taken for promoter analysis. A 5kb upstream sequence from the transcription start site of each of these genes was retrieved and the binding sites of transcription factors within this region were determined as described above. Table 3 is a list of the transcription factor binding sites in a set of clustered genes within the indicated GO term. Significant transcription factors consistently occurring in the promoters of genes altered by TNFα and clustering together with common GO terms included SREBP-1, CEBPα, MEF2 and AREB6. All these are in several ways implicated in cellular metabolic processes and immune response, two phenomena that are modulated by TNFα. Surprisingly, binding sites of several members of the forkhead family of transcription factors, that are however not well characterised were also significantly found to be overrepresented in our dataset. These included FOXJ2, FOXD3 and the *fork head* homolog-3 (HFH-3). The biological relevance of these relatively less characterized transcription factors remain to be investigated.

**Table 3:** Over representation of conserved transcription factor binding sites within putatively co-regulated genes.

GO term and Gene Symbol*	Transcription Factors	Probability**
***Metabolism***		
SQLE, EBP, TIMP1, DHRS4, UBE2J1,	FOXJ2	3.31E-156
UBE2NL, PAH, HSP90B1, GCDH	FOXD3	5.75E-136
DHFR, FADS, SIRT6, AKR1B1	HFH-3	2.42E-128
C4B, SLC25A5	IRF-1	4.06E-108
	SREBP-1	1.41E-53
	E47	1.17E-52
	MEF-2	6.67E-42
	AREB6	9.87E-41
	ARP-1	1.51E-35
***Signal Transduction***		
TBL1X, RYK, DDX54, TNFRSF14,	FOXJ2	3.04E-58
KISSIR, FCER1G, NFkB2	FOXD3	5.50E-58
	IRF-1	3.42E-52
	HFH-3	8.90E-48
	SREBP-1	2.71E-19
	CHOP:C/EBPalpha	2.51E-14
	ARP-1	1.26E-13
	Freac-7	2.98E-12
	E47	2.78E-11
	MEF-2	5.14E-11
***Regulation of biological processess***		
CASZ1, PUM1, C4B, GTF2H3	MEF-2	1.06E-14
ATF5,	CHOP:C/EBPalpha	1.93E-14
	FOXJ2	6.57E-13
	AREB6	3.25E-12
	SREBP-1	3.96E-12
	ARP-1	1.69E-11
	p300	2.32E-11
	FOXD3	3.39E-11
	SREBP-1	3.09E-10
	NF-E2	3.89E-10

*Genes that clustered together and occurred within the same GO term.

**Significant probability of the binding of the respective transcription factors to the indicated sets of genes.

### EMSA for Validation of Candidate Transcription Factors

**Figure 4:**
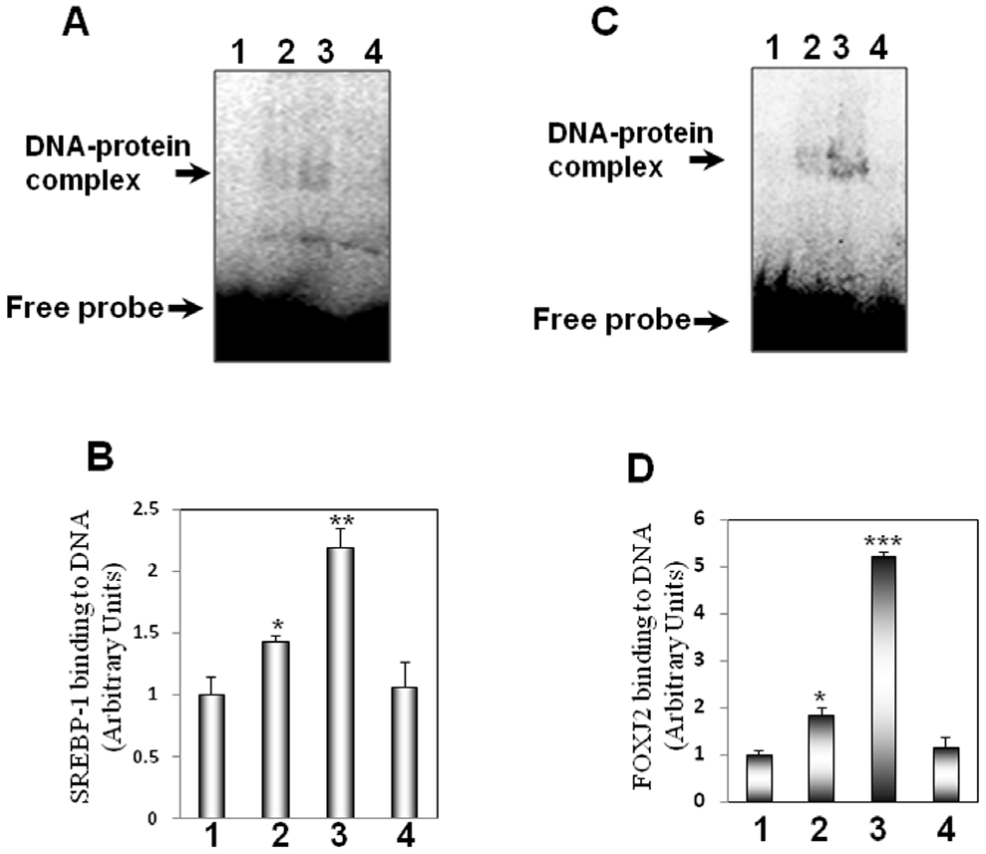
Electrophoretic mobility shift assay in control and TNFα-treated nuclear extracts using SREBP-1 and FOXJ2 oligonucleotide probes. 15 µg of nuclear extracts from control or TNFα-treated cells were incubated with labeled probes harboring the binding elements of either SREBP-1 (A) or FOXJ2 (C). On termination of incubation they were resolved in a non-denaturing polyarylamide gel and subjected to phosphorimager analysis. Mutated oligonucleotides with altered binding motifs of these two transcription factors were used to check the specificity of the DNA-protein complexes. All experiments were done in triplicate. B and D: Densitometric analyses of the binding of SREBP-1 (B) and FOXJ2 (D) respectively. Each point is the mean±SEM of three independent experiments. Lane 1: Labeled probe alone; Lane 2: Labeled probe incubated with nuclear extract from control cells; Lane 3: Labeled probe incubated with nuclear extract from TNFα-treated cells; Lane 4: Labeled mutated probe incubated with the nuclear extract from control cells. *p<0.05 as compared to Lane 1; **p<0.01 as compared to Lanes 1 and 2; ***p<0.001 as compared to Lanes 1 and 2.

To validate the transcription factors that were found to possess binding sites in the promoters of genes that were altered by TNFα as described above, electrophretic mobility shift assay was performed for two of the candidate transcription factors namely, SREBP-1 and FOXJ2. Wild type and mutated oligonucleotide sequences that were used are given in Table 4. As compared to the incubation in the presence of the free labeled probe alone, the addition of the nuclear extract from the control cells caused significant formation of the DNA-protein complex both with SREBP-1 (Figure 4A, B) and FOXJ2 (Figure 4C, D) oligonucleotides. This complex formation was significantly enhanced in the presence of nuclear extracts from TNFα-treated cells indicating that TNFα increased the binding of these transcription factors to their binding elements that corroborates with our predicted findings detailed above. No detectable DNA-protein complexes were identified with the mutated oligonucleotides of either SREBP-1 or FOXJ2 that additionally suggests the specificity of the DNA-protein complexes observed.

**Table 4:** Sequences for the binding elements and their mutated counterparts used in the EMSA analysis.

SREBP-1 FP	5′ GATCCTGATCACCCCACTGAGGAG 3′
SREBP-1 RP	5′ CTCCTCAGTGGGGTGATCAGGATC 3′
SREBP-1 mutated FP	5′ GATCCTGATC**T**CCC**G**ACTGAGGAG 3′
SREBP-1 mutated RP	5′ CTCCTCAGT**C**GGG**A**GATCAGGATC 3′
FOXJ2 FP	5′ CACATAAACAAATACATCA3′
FOXJ2 RP	5′ TGATGTATTTGTTTATGTG 3′
FOXJ2 mutated FP	5′ CAC**G**TAA**G**CAAATACATCA 3′
FOXJ2 mutated RP	5′ TGATGTATTTG**C**TTA**C**GTG 3′

FP: Forward Primer.

RP: Reverse Primer.

Poistions of alterations in the mutated oligonucleotides within the binding motifs are shown by bold and underlined bases.

### Network and Pathway Analyses

**Figure 5:**
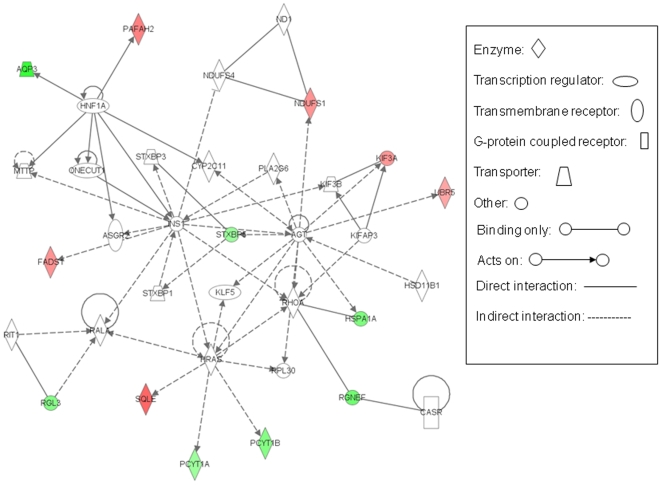
Interacting network among genes altered by TNFα in HepG2 cells. Functional interacting network among genes altered with a log_2_ ratio of ±0.5 in HepG2 cells by TNFα. All such genes were uploaded in the Ingenuity Pathway Analysis tool and the network of “lipid metabolism, small molecule biochemistry, nervous system development and function” that was identified with a significant score is depicted. Genes from our dataset and falling in this network are shown in either red (up-regulated) or green (down regulated) with the intensity of the color being an indicator of the fold change.

We then wanted to identify the interacting network among the altered genes in the context of other large biological pathways. We began by analyzing TNFα-induced altered genes using the Ingenuity Pathway Analysis software that generates networks, associated biological functions and canonical pathways by overlaying them onto a global molecular network developed from information contained in the Ingenuity knowledge base. Five highly significant networks with scores above 20 were obtained from the set of TNFα induced altered genes. These scores, derived from p values, indicated the likelihood of the focus genes belonging to a network versus those obtained by chance alone thereby eliminating the probability of their occurrence in a network to be due to noise. The network with the highest score comprised that of hematological system development and function, immune and lymphatic system development and function and tissue morphology. Other subsequent networks with decreasing scores included those of (i) immunological disease, skeletal and muscular disorders and inflammatory disease (ii) lipid metabolism, small molecule biochemistry, nervous system development and function, (iii) cell signaling, post translational modification, dermatological diseases and conditions and (iv) cellular growth and proliferation, cancer and hematological disease. This implies that the altered genes were distributed among diverse networks that could be expected considering the varied cellular actions of TNFα. Interestingly, while “biosynthesis of steroids” was the top canonical pathway that was significantly (with a p value of 1.27E-03) altered by TNFα in HepG2 cells, “lipid metabolism” was the most significant (1.70E-04-4.39E-02) molecular and cellular function to be affected. So we further analyzed the network 3 of the list of significantly affected networks i.e of lipid metabolism, small molecule biochemistry, nervous system development and function since it could also be correlated well with the most significantly affected canonical pathway and molecular and cellular function. Figure 5 shows the associated network functions of this interaction. One of the key central points in this network is that of insulin (INS1). Apart from regulating FADS1, INS1 indirectly also regulates HRAS that inturn regulates SQLE, these are the two significantly upregulated genes in our dataset. Insulin was postulated some time ago by Satoh et al., 1990 [38] to regulate SQLE and insulin regulation of hepatic desaturases has also been described [39]. INS1 is directly regulated by HNF1A that in turn interacts with AQP3 and PAFAH2 that were down and up regulated, respectively, in our experiment. HRAS shows interactions with SQLE, PCYT1A and 1B, genes that were differentially regulated by TNFα. Another central point in this interaction network was occupied by RHOA (ras homolog gene family, member A). Two of the significantly altered genes from our list, HSPA1A and RGNEF, were found to be binding strongly with RHOA. Interestingly, RHOA is also regulated by HRAS on one end and INS1 on the other, although indirectly. All these indicate a cross talk among molecules that otherwise seem to be distantly placed in biological pathways and therefore many positions of this network being occupied by genes altered by TNFα possibly explains to some extent the diverse effects of TNFα on cellular physiology.

**Figure 6:**
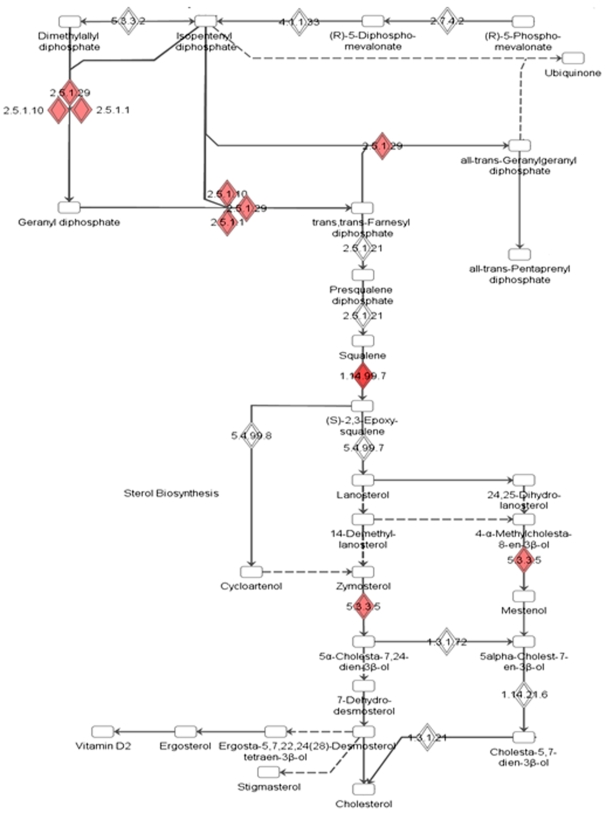
“Biosynthesis of steroids” identified as the top canonical pathway altered by TNFα in HepG2 cells. Significant positions of the pathway are occupied by genes from our dataset indicating that this pathway is invariably affected by TNFα in HepG2 cells. These positions have been marked with solid diamond boxes in the figure. All the genes clustered at this part of the complete “Biosynthesis of steroids” pathway and therefore are indicative of the role of TNFα in the regulation of this pathway within the hepatocyte. 1.14.99.7: Squalene Epoxidase; 5.3.3.5: Emopamil Binding Protein (sterol isomerase); 2.5.1.1, 2.5.1.10, 2.5.1.129: forms of Farnesyl Diphosphate Synthase.

**Figure 7:**
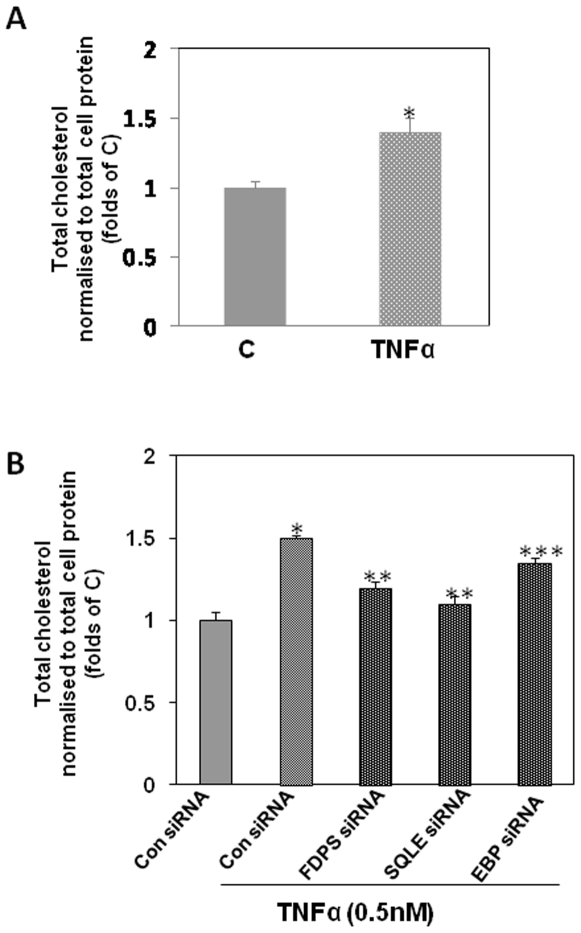
Effect of TNFα on cholesterol synthesis in HepG2 cells. A.HepG2 cells were incubated in the absence (C) or presence of TNFα (0.5 nM, 12 h) and cells were lysed and lipids were extracted by addition of 1.0 ml of chloroform: methanol (2:1). B. HepG2 cells were transfected with 50 nM of either control (Con siRNA) or SQLE or FDPS or EBP siRNAs. After 48 h, they were incubated either alone or in the presence of TNFα as in “A”. Cholesterol was estimated in the extracted lipid fraction as described in the Methods section. Results are expressed after normalization to the total protein content. Each value is the mean± SEM of three experiments. *p<0.05 as compared to control (C), determined using Student's *t* test (A). *p<0.001 as compared to ConsiRNA; **p<0.01 and ***p<0.05 as compared to the incubation of TNFα alone in the presence of ConsiRNA (B).

Figure 6 shows the “biosynthesis of steroids” pathway obtained using the Ingenuity Pathway Analysis tool that was derived as the most significant pathway being affected by our set of TNFα induced altered genes. Most of the affected genes clustered at significant points of the cholesterol biosynthetic pathway. These include three isoforms of farnesyl diphosphate synthase (FDPS, EC nos. 2.5.1.1; 2.5.1.10; 2.5.1.29), squalene expoxidase (SQLE, EC no. 1.14.99.7) and emopamil binding protein (EBP, sterol isomerase, EC. no. 5.3.3.5). All these were upregulated and have been marked with red diamond boxes in the figure with the intensity of the color being an indicator of the fold of up-regulation. Two of these namely SQLE and FDPS were also validated by Real-Time PCR (Figure 2). Phenotypically, as a consequence of this, we observed enhanced cholesterol accumulation in HepG2 cells on treatment with TNFα (0.5 nM) for 12 h (Figure 7A). In an attempt to understand which of the above TNFα-altered genes of this pathway is the most significant contributor for the observed increase in the cholesterol levels in the presence of TNFα, siRNAs were used to knock down the specific genes namely SQLE, FDPS and EBP. Use of the siRNAs could knock down the levels of the respective proteins by 75% (data not shown). Figure 7B shows the effect of TNFα on the cellular cholesterol levels in the presence of the siRNAs. In cells transfected with SQLE or FDPS siRNAs, the TNFα-mediated increase in the cellular cholesterol content was significantly (p<0.01) prevented with SQLE siRNA demonstrating a higher fold of prevention. EBP siRNA however depicted a very mild preventive effect. All these indicate that of these three enzymes of the steroid biosynthetic pathway that are up-regulated by TNFα, SQLE is the most significant contributor of the elevated synthesis of cholesterol by TNFα in HepG2 cells. This pathway emerging as the top canonical pathway is therefore indicative of a correlation between cellular lipid homeostasis and the inflammatory molecule that may possibly underlie the pathogenesis of several diseases.

## Discussion

TNFα is a major proinflammatory cytokine that activates varied cellular signaling pathways resulting in diverse biological responses. Within the liver, TNFα is known to regulate contrasting processes. On the one hand, during liver regeneration for e.g.after partial hepatectomy TNFα induces hepatocyte proliferation thereby regulating normal cell function while on the other hand, in the presence of any hepatocyte injury, TNFα production can also trigger cell death rather than proliferation. TNFα therefore is a crucial deciding factor between hepatocyte life and death and therefore of global liver homeostasis. In the context of these paradoxically contrasting hepatic responses towards TNFα, we employed high density oligonucleotide arrays (Human Genome U133 plus 2.0, Affymetrix) to identify hepatic genes altered by TNFα and elucidate the common transcriptional regulatory controls among the altered genes.

Microarray analysis of HepG2 genes altered by TNFα identified 67 genes to be up- and 73 genes to be down-regulated. Very significantly and consistently altered were genes of lipid metabolism mainly squalene epoxidase, fatty acid desaturase, farnesyl diphosphate synthase and hydroxysteroid (17-β) dehydrogenase 7 thus indicating lipid metabolism to be crucially targeted by TNFα. This suitably agrees with the fact that TNFα is very significant in the pathogenesis of fatty liver diseases that accompanies a host of metabolic disorders. TNFα has been shown to induce fat accumulation in the liver resulting in hepatic steatosis [40]. In fact, Ruan et al., [41] also reported that squalene epoxidase and farnesyl diphosphate synthase were increased by 2.4 and 1.7 fold respectively in the rat liver on infusion with TNFα. The transcription factor, CEBPα is implicated in the regulation of numerous liver-specific genes and it is a critical regulator of hepatic metabolism [42]–[46] and our results are in agreement with earlier reports [47], [48] that show a significant inhibition of CEBPα by TNFα thereby implying a major alteration of cellular metabolism.

The transcription factor, ATF5 binds to the cAMP response element (CRE) on cAMP inducible promoters and regulates several hepatic specific enzymes [49] and its loss accompanies hepatic carcinomas [50]. Not reported earlier, our demonstration of ATF5 being regulated by TNFα in HepG2 cells, therefore, is indicative of its participation in the cellular role of TNFα in hepatic physiology. Our results also demonstrated that genes involved in cell death and cell proliferation were significantly altered by TNFα. Earlier reports also demonstrated the same with the transcription factor NF-κB and c-jun playing a significant mediatory role therein [51], [52]. TNFα acts primarily through the NFκB and MAPK pathways and their activation is observed immediately as initiators of downstream signaling cascades. In our study, we observed activation of two downstream molecules of these pathways namely, NFKB2 (Nuclear Factor of Kappa light Polypeptide Gene Enhancer in B-cells 2) and MKNK2 (MAPK interacting serine/threonine kinase 2). NFKB2 is a subunit of NFκB that has been shown to be increased at the mRNA and protein level by TNFα [53] and its inappropriate activation is linked to varied pathophysiological states. MKNK2 contains a MAPK binding and activating motif and it participates in the regulation of protein biosynthesis and translation, in the protein kinase cascade and in response to stress. The involvement of the MAPK pathway in TNFα-induced toxic liver injury was reported in an in vivo mouse model [54] and our study has demonstrated an up-regulation of MKNK2 by 1.8-fold in HepG2 cells by TNFα. Also, MKNK2 is known to interact with TRAF2 (TNF receptor-associated factor 2) and thereby play a significant role in TNFα receptor activity associated cellular phenomena.

Genes involved in “Cell Adhesion” were significantly down regulated implying a compromise in these phenomena by TNFα within the hepatocyte. In our study we found laminin gamma 1 (LAMC1) and TIMP 1 to be significantly down regulated (−1.4 fold and −1.5 fold respectively) by TNFα indicating a modulation of the extracellular matrix. The regulation of laminin, TIMP1 and MMP9 by TNFα has been described [55]–[59] and here our results show decreased expression of TIMP1 that together with the decreased laminin gamma 1 expression indicate towards a modulation of the extracellular matrix and thereby of cell adhesion within the hepatocyte by TNFα.

Interestingly, in the present study, a catalog of genes not earlier reported to be regulated by TNFα were found to be altered in HepG2 cells. Significant among these were those from the GO term: nucleic acid metabolism mainly DHFR, MTHFS, SIRT6 and DNMT3B; almost all of these are involved in the synthesis, degradation or function of nucleic acids. Nucleic acids and their metabolites are critical in the elucidation of an immune response [60] and the alteration of these genes by TNFα as identified in our study could at least in part possibly explain the role of nucleic acids in the effects of TNFα in HepG2 cells. Also several altered genes that categorized within the GO term “Signal transduction” like RASSF7, RGL3, KISSIR and RYK are being reported here for the first time to be regulated by TNFα. RASSF7 and RGL3 are significant signaling intermediates of the RAS pathway and their alterations indicate towards modulation of this important cellular signaling cascade by TNFα in HepG2 cells. Another important molecule affected by TNFα is the gene RYK receptor like tyrosine kinase (RYK) that like RTKs are deregulated in cancers [61] and our revelation of the regulation of its gene expression by TNFα in HepG2 cells could provide information surrounding these phenomena. Our study, therefore, apart from demonstrating the effects of TNFα on genes already known to be regulated as such, has also unearthed several genes that were hitherto not known to be regulated by TNFα.

Several well characterized and other not so well characterized transcription factors were identified with binding elements in sets of co-regulated genes. Myocyte enhancer factor 2 or MEF-2 family of transcription factors that bind as homo- and heterodimers to the MEF2 sites in the promoter regions of target genes were found to be the most enriched in genes that belonged to the “Regulation of Biological Processes” GO term (Table 3) suggesting that this may be a major determiner of these processes within the hepatocyte. Sterol Regulatory Element Binding Proteins (SREBPs) are a basic-helix-loop-helix leucine zipper class of transcription factors that bind to the sterol regulatory element DNA sequence TCACNCCAC. Within the liver, this transcription factor is known to regulate several hepatic processes mainly gluconeogenesis [62], lipid and cholesterol biosynthesis [63] and thereby attains significance in diverse metabolic disturbances. TNFα has been shown to stimulate the maturation of SREBP-1 in hepatocytes [64] and our results demonstrated herein imply that SREBP-1 could be important in mediating the effects of TNFα on these genes.

Interferon regulatory factor 1 or IRF-1 is a member of the interferon regulatory transcription factor (IRF) family and activates interferon transcription as well as of genes induced by interferons [65]. IRF1 exhibits regulatory roles in diverse cellular processess [66], [67] and it has been associated with some forms of cancer mainly lung and gastric cancer. Our demonstration of IRF-1 depicting a high probability of over-representation in genes that belonged to the “Metabolism” and “Signal Transduction” GO termsindicates that this factor might modulate genes involved with these GO terms and thereby underlie the crosstalk between inflammation and cell cycle phenomena on the one end and general cellular metabolism on the other.

Other than these, the less well characterized but emerging to be possessing significant binding elements in our dataset include FOXD3, FOXJ2 and HFH-3. These belong to the forkhead box proteins (FOX proteins) that were initially known as regulators of embryonic development and have now been recognized to play important roles in regulating the expression of genes involved in cell growth, proliferation and differentiation. FOXD3 has recently been shown to be involved in the maintenance of neural crest and pluripotent cells [68], [69]. The expression of FOXJ2 starts early in life and continues till adulthood. It binds to two different DNA binding sites [70] and our depiction of its enhanced binding to its element in the presence of TNFα suggests that it might be critical in the TNFα action in HepG2 cells. The hepatocyte nuclear factor-3 (HNF-3) *fork head* homolog (HFH) proteins share homology to the winged helix DNA binding domain and have been implicated in cell development and organogenesis [71]. Study of the role of these factors in the context of various cellular processes, alterations of which, underlie the pathogenesis of several diseases is an area worthy of investigation.

One of the most significant networks that emerged from the TNFα induced altered genes was that of lipid metabolism and this subsequently correlated well with the top canonical pathway, the biosynthesis of steroids. TNFα increases hepatic cholesterol synthesis [72] and interferes with lipid homeostasis [73]. Modulation of SQLE and FDPS has been shown to alter intracellular hepatic cholesterol synthesis [74]–[76] implying their significance in this pathway. Also, SREBP-1 has been demonstrated to regulate the expression of these genes in HepG2 cells and the liver [77], [78] that also additionally validates our transcription factor predictions and analyses. In our study, TNFα significantly elevated intracellular cholesterol levels in HepG2 cells which validates our claim of this pathway being the most favored and in these series of events, SQLE is the most significant mediator of the effects of TNFα on cholesterol synthesis. TNFα induces hepatic steatosis in mice [40] with increased hepatic lipid synthesis [72], [79] **.** Such an effect of TNFα on hepatic lipid homeostasis was also reported recently by Qin *et al.* [80] where they demonstrated that in vivo infusion of TNFα significantly increased hepatic production of total circulatory apolipoprotein B100 and VLDL apolipoprotein B100 in fasting and post prandial states in a hamster model.

To conclude, our results demonstrate that TNFα induced alteration in the hepatocyte transcriptome majorly includes genes of lipid metabolism that acutely targets biosynthesis of steroids and cholesterol. While several characterized transcription factors were consistently found to be having binding elements in these sets of genes, others mainly those of the fork head family that are not too well characterized also appeared to be substantially over represented. Our results presented here put forth an overall detailed analysis of the action of TNFα in HepG2 cells by encompassing the altered gene expression profile, the different GO functional terms these altered genes categorise in and the consequent predicted signatures of conserved transcription factor binding sites in these sets of genes. Given that TNFα is now invariably being associated with several diseases, our results provide information towards identification of the underlying genes and transcription factors that determine among other significant liver phenotypes, hepatic steatosis and fatty liver that are critically associated with several pathological states.

## Supporting Information

Figure S1 Dose and time dependent effect of TNFα on insulin stimulated Akt phosphorylation in HepG2 cells.
(0.11 MB TIF)

Figure S2 Hierarchical cluster of the differentially expressed genes.
(0.31 MB TIF)

Table S1 Genes altered by TNFα treatment in HepG2 cells.
(0.19 MB DOC)

Table S2 Gene Ontology (Biological Processes) terms overrepresented (p<0.01) in the set of genes altered by TNFα.
(0.23 MB DOC)
