# Supplementary figures and images for: Gene Expression Profiling and Network Analysis Reveals Lipid and Steroid Metabolism to Be the Most Favored by TNFα in HepG2 Cells
Source: PLoS One. 2010 Feb 4;5(2):e9063. doi: 10.1371/journal.pone.0009063 (PMC2816217; doi:10.1371/journal.pone.0009063)

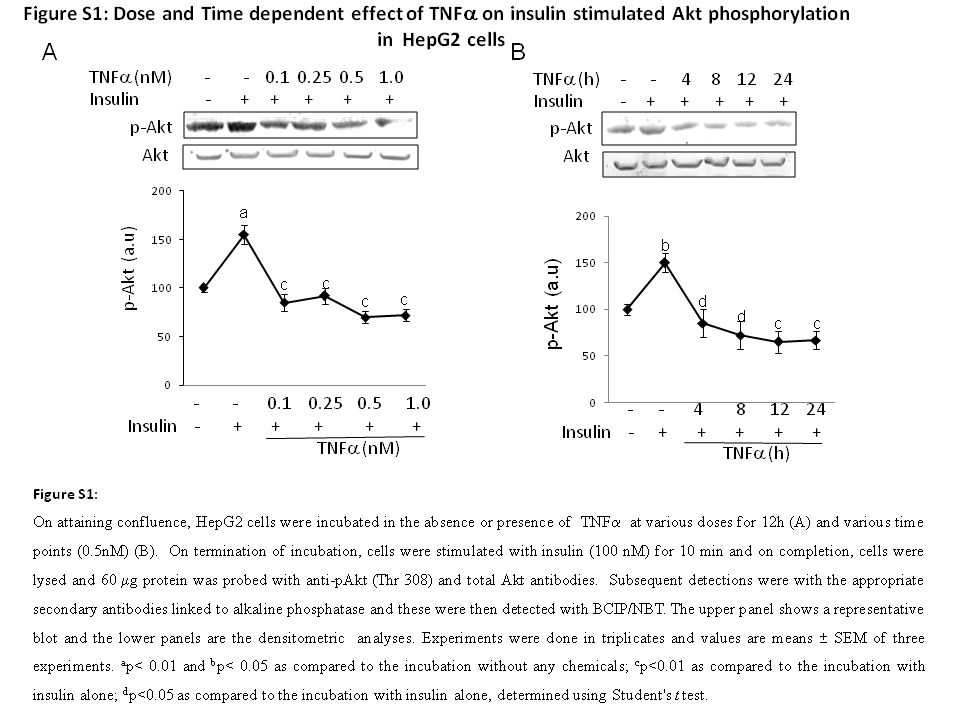

Supplement: Figure S1 — Dose and time dependent effect of TNFα on insulin stimulated Akt phosphorylation in HepG2 cells. (0.11 MB TIF) [file pone.0009063.s001.tif]

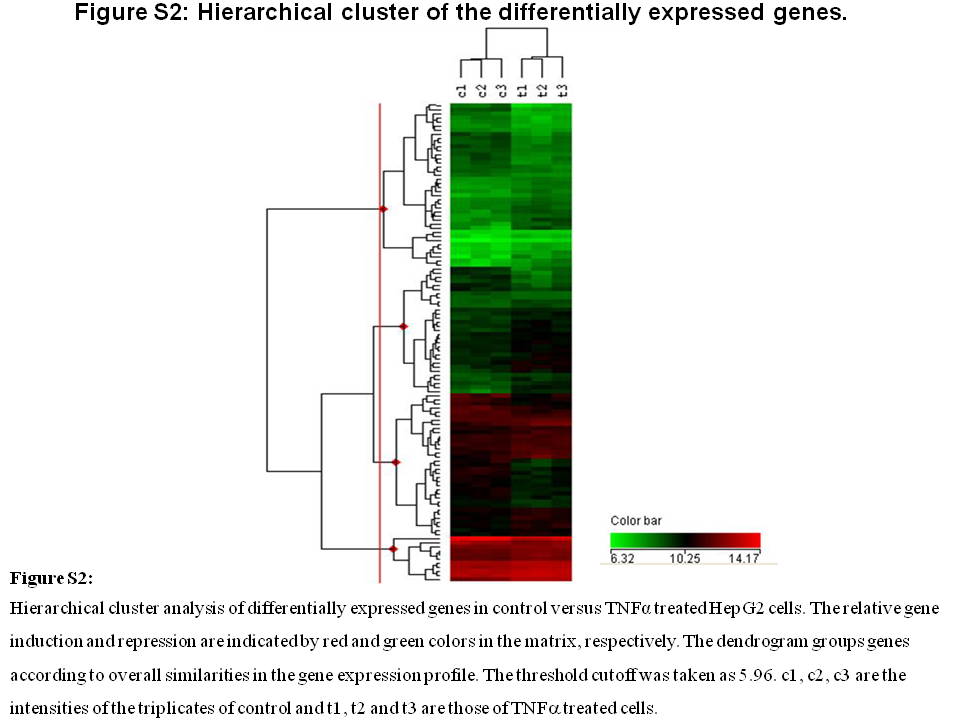

Supplement: Figure S2 — Hierarchical cluster of the differentially expressed genes. (0.31 MB TIF) [file pone.0009063.s002.tif]
