# Supplementary material for: Gene Expression Profiling and Network Analysis Reveals Lipid and Steroid Metabolism to Be the Most Favored by TNFα in HepG2 Cells
Source: PLoS One. 2010 Feb 4;5(2):e9063. doi: 10.1371/journal.pone.0009063 (PMC2816217; doi:10.1371/journal.pone.0009063)
Supplement: Table S1 — Genes altered by TNFα treatment in HepG2 cells. (0.19 MB DOC) [file pone.0009063.s003.doc]

**Table S1**. Genes altered by TNFα treatment in HepG2 cells.

| **Gene Symbol** | **Fold Change** | **Gene ID** | **Description** | **p Val** |
| --- | --- | --- | --- | --- |
| ***Cell adhesion*** | |  |  |  |
| LAMC1 | -1.4 | 200770_s_at | Laminin, Gamma 1 (Formerly Lamb2) | 0.01 |
| TAOK2 | -1.6 | 204877_s_at | Tao Kinase 2 | 0.04 |
| FGFRL1 | -1.5 | 223321_s_at | Fibroblast Growth Factor Receptor-Like 1 | 0.03 |
| DGCR6L | 1.4 | 208024_s_at | Digeorge Syndrome Critical Region Gene 6-Like | 0.04 |
| CXADR | 1.5 | 226374_at | Coxsackie Virus And Adenovirus Receptor | 0.03 |
| ***Regulation of biological process*** | | |  |  |
| PUM1 | -1.8 | 201165_s_at | Pumilio Homolog 1 (Drosophila) | 0.03 |
| CEBPA | -1.7 | 204039_at | CCAAT/Enhancer Binding Protein (C/EBP), Alpha | 0.006 |
| BRPF1 | -1.6 | 204481_at | Bromodomain And PHD Finger Containing, 1 | 0.04 |
| STARD10 | 1.5 | 232322_x_at | Start Domain Containing 10 | 0.04 |
| CASZ1 | 1.5 | 220015_at | Castor Homolog 1, Zinc Finger (Drosophila) | 0.03 |
| GTF2H3 | 1.5 | 222104_x_at | General Transcription Factor LJH, | 0.03 |
|  |  |  | Polypeptide 3, 34kDa |  |
| ATF5 | -1.5 | 204998_s_at | Activating Transcription Factor 5 | 0.01 |
| SAPS3 | 1.7 | 1556590_s_at | Chromosome 11 Open Reading Frame 23 | 0.02 |
| ***Signal transduction*** | |  |  |  |
| FCER1G | -1.6 | 204232_at | Fc Fragment Of IgE, High Affinity I, | 0.03 |
|  |  |  | Receptor For; Gamma Polypeptide |  |
| RASSF7 | -1.7 | 204927_at | Ras Association (RALGDS/AF-6) Domain Family7 | 0.04 |
| NR1H4 | -1.7 | 206340_at | Nuclear Receptor Subfamily 1, Group H, | 0.009 |
|  |  |  | Member 4 |  |
| MKNK2 | 1.8 | 223199_at | MAP Kinase Interacting Serine/Threonine | 0.005 |
|  |  |  | Kinase 2 |  |
| RGL3 | -1.8 | 228877_at | Ral Guanine Nucleotide Dissociation | 0.01 |
|  |  |  | Stimulator-Like 3 |  |
| TBL1X | 1.7 | 201867_s_at | Transducin (Beta)-Like 1X-Linked | 0.03 |
| RYK | 1.6 | 202853_s_at | RYK Receptor-Like Tyrosine Kinase | 0.04 |
| HIST1H4B | 1.9 | 205967_at | H4 Histone, Family 2 | 0.01 |
| NFKB2 | 1.5 | 209636_at | Nuclear Factor Of Kappa Light Polypeptide | 0.002 |
|  |  |  | Gene Enhancer In B-Cells 2 |  |
| DDX54 | 1.5 | 219111_s_at | DEAD (Asp-Glu-Ala-Asp) Box Polypeptide 54 | 0.01 |
| IL22RA1 | 1.5 | 220056_at | Interleukin 22 Receptor, Alpha 1 | 0.03 |
| GSG2 | 1.6 | 223759_s_at | Germ Cell Associated 2 (Haspin) | 0.04 |
| TNFRSF14 | 1.5 | 209354_at | Tumor Necrosis Factor Receptor Super | 0.02 |
|  |  |  | Family,Member14(Herpesvirus Entry Mediator) |  |
| THRAP6 | 1.7 | 227786_at | Thyroid Hormone Receptor Associated Protein 6 | 0.03 |
| KISS1R | 1.7 | 242517_at | KISS1 Receptor | 0.01 |
| ***Development*** |  |  |  |  |
| TGIF | -1.4 | 1566901_at | TGFB-Induced Factor (Tale Family Homeobox) | 0.03 |
| JRKL | 1.8 | 206734_at | Jerky Homolog-Like (Mouse) | 0.04 |
| ***Response to stimulus*** | |  |  |  |
| HSPA1B | -1.9 | 200799_at | Heat Shock 70kDa Protein 1A | 0.01 |
| ORM2 | -1.5 | 214465_at | Orosomucoid 2 | 0.005 |
| MST1 | -1.5 | 216320_x_at | Macrophage Stimulating 1 (Hepatocyte | 0.02 |
|  |  |  | Growth Factor-Like) |  |
|  |  |  |  |  |
| **Gene Symbol** | **Fold Change** | **Gene ID** | **Description** | **p Val** |
| H3/O | -2 | 222067_x_at | Histone 1, H2BD | 0.01 |
| C4B | -1.5 | 235856_at | Complement Component 4A (Rodgers | 0.02 |
|  |  |  | Blood Group) |  |
| ***Death*** |  |  |  |  |
| TM2D1 | -2.4 | 236524_at | TM2 Domain Containing 1 | 0.009 |
| PDCD7 | 1.4 | 231809_x_at | Programmed Cell Death 7 | 0.02 |
| ***Transport*** |  |  |  |  |
| FLJ11506 | -2 | 202852_s_at | Hypothetical Protein Flj11506 | 0.04 |
| AP3B1 | -1.7 | 203141_s_at | Adaptor-Related Protein Complex 3, Beta1 Subunit | 0.03 |
| SLC6A12 | -3.2 | 206058_at | Solute Carrier Family 6 (Neurotransmitter | 0.04 |
|  |  |  | Transporter, Betaine/GABA), Member 12 |  |
| RAB11FIP1 | -1.8 | 219681_s_at | Rab11 Family Interacting Protein 1 (Class I) | 0.04 |
| STXBP5 | -1.5 | 226794_at | Syntaxin Binding Protein 5 (Tomosyn) | 0.03 |
| AP1S2 | -1.4 | 228415_at | Adaptor-Related Protein Complex 1, Sigma2 | 0.04 |
|  |  |  | Subunit |  |
| AQP3 | -2.9 | 39248_at | Aquaporin 3 (Gill Blood Group) | 0.02 |
| LOC442285 | 1.5 | 1553148_a_at | Hypothetical LOC442285 | 0.003 |
| SRGAP2 | 1.7 | 1568957_x_at | Slit-Robo Rho GTPase Activating Protein 2 | 0.04 |
| GOSR2 | 1.4 | 210009_s_at | Golgi Snap Receptor Complex Member 2 | 0.02 |
| SEC24A | 1.4 | 212900_at | Sec24 Related Gene Family, Member A | 0.03 |
|  |  |  | (S. Cerevisiae) |  |
| HCN3 | 1.7 | 222078_at | Hyperpolarization Activated Cyclic | 0.03 |
|  |  |  | Nucleotide-Gated Potassium Channel 3 |  |
| SLC43A2 | 1.9 | 226629_at | Solute Carrier Family 43, Member 2 | 0.04 |
| FLJ38991 | 1.4 | 227134_at | Synaptotagmin-Like 1 | 0.02 |
| VPS41 | 1.7 | 235625_at | Vacuolar Protein Sorting 41 (Yeast) | 0.003 |
| C6ORF69 | 1.6 | 228299_at | Chromosome 6 Open Reading Frame 69 | 0.01 |
| KIF3A | 1.7 | 228680_at | Kinesin Family Member 3A | 0.04 |
| ***Cell proliferation*** | |  |  |  |
| TIMP1 | -1.5 | 201666_at | TIMP Metallopeptidase Inhibitor 1 | 0.04 |
| EDD1 | 1.5 | 1555888_at | E3 Ubiquitin Protein Ligase, Hect Domain | 0.01 |
|  |  |  | Containing, 1 |  |
| NRD1 | 2.1 | 242235_x_at | Nardilysin (N-Arginine Dibasic Convertase) | 0.005 |
| ***Homeostasis*** |  |  |  |  |
| PDIA4 | -1.9 | 208658_at | Protein Disulfide Isomerase Family A,Member4 | 0.01 |
| PDIA4 | -1.9 | 211048_s_at | Protein Disulfide Isomerase Family A,Member4 | 0.03 |
| CALR | -1.6 | 214315_x_at | Calreticulin | 0.002 |
| ***Gene expression*** | |  |  |  |
| POLR2J3 | -1.8 | 1552621_at | DNA Directed RNA Polymerase II Polypeptide | 0.03 |
|  |  |  | J-Related Gene |  |
| LOC113179 | -1.6 | 1553968_a_at | Hypothetical Protein BC011824 | 6.9E-05 |
| RGNEF | -2.1 | 1554003_at | Rho-Guanine Nucleotide Exchange Factor | 0.03 |
| ATF7IP2 | -1.9 | 228381_at | Activating Transcription Factor 7 | 0.008 |
|  |  |  | Interacting Protein 2 |  |
| LOC148203 | -1.5 | 229700_at | Hypothetical Protein LOC148203 | 0.03 |
| SLA/LP | -1.7 | 231730_at | Soluble Liver Antigen/Liver Pancreas Antigen | 0.04 |
| C14ORF172 | -1.6 | 52741_at | Chromosome 14 Open Reading Frame 172 | 0.03 |
|  |  |  |  |  |
|  |  |  |  |  |
| **Gene Symbol** | **Fold Change** | **Gene ID** | **Description** | **p Val** |
| ***Protein metabolism*** | |  |  |  |
| STOM | -1.7 | 201061_s_at | Stomatin | 0.03 |
| UBE2J1 | -1.6 | 222435_s_at | Ubiquitin-Conjugating Enzyme E2,J1(UBC6 | 0.03 |
|  |  |  | Homolog,Yeast) |  |
| SCRN3 | -1.5 | 222849_s_at | Secernin 3 | 0.04 |
| 7-Sep | 1.7 | 1565823_at | Septin 7 | 0.03 |
| TRIM5 | 2 | 210705_s_at | Tripartite Motif-Containing 5 | 0.03 |
| UBE2NL | 1.7 | 217393_x_at | Ubiquitin-Conjugating Enzyme E2N-Like | 0.04 |
| PSME3 | -1.9 | 200987_x_at | Proteasome (Prosome, Macropain) | 0.04 |
|  |  |  | Activator Subunit 3 (PA28 Gamma; Ki) |  |
| HSP90B1 | -2.1 | 216449_x_at | Heat Shock Protein 90kDa Beta (Grp94), | 0.03 |
|  |  |  | Member 1 |  |
| IMPACT | 1.6 | 218637_at | Impact Homolog (Mouse) | 0.03 |
| ***Carbohydrate metabolism*** | |  |  |  |
| AKR1B1 | -1.4 | 201272_at | Aldo-Keto Reductase Family 1, Member B1 | 0.03 |
|  |  |  | (Aldose Reductase) |  |
| GNPDA2 | -1.8 | 227022_at | Glucosamine-6-Phosphate Deaminase 2 | 0.04 |
| PDK3 | -1.8 | 230085_at | Pyruvate Dehydrogenase Kinase, Isozyme 3 | 0.04 |
| IDH2 | 1.5 | 210046_s_at | Isocitrate dehydrogenase 2 (NADP+), Mito | 0.03 |
| ***Amino acid metabolism*** | |  |  |  |
| C1ORF69 | -1.6 | 236629_at | Chromosome 1 Open Reading Frame 69 | 0.01 |
| PAH | 1.8 | 242375_x_at | Phenylalanine Hydroxylase | 0.03 |
| ***Nucleic Acid metabolism*** | |  |  |  |
| MTHFS | 1.7 | 210242_x_at | 5,10-Methenyltetrahydrofolate Synthetase | 0.03 |
|  |  |  | (5-Formyltetrahydrofolate Cyclo-Ligase) |  |
| EP400 | -1.5 | 212376_s_at | Trinucleotide Repeat Containing 12 | 0.04 |
| SIRT6 | -1.5 | 219613_s_at | Sirtuin (Silent Mating Type Information | 0.03 |
|  |  |  | Regulation 2 Homolog) 6 (S. Cerevisiae) |  |
| ELF5 | -1.7 | 220625_s_at | E74-Like Factor 5 (ETS Domain Transcription | 0.04 |
|  |  |  | Factor) |  |
| DNMT3B | -1.9 | 220668_s_at | DNA (Cytosine-5-)-Methyltransferase 3 Beta | 0.01 |
| NDUFS1 | 1.7 | 1559691_at | NADH Dehydrogenase (Ubiquinone) Fe-S | 0.02 |
|  |  |  | Protein 1, 75kDa (NADH-Coenzyme Q Reductase) | |
| DHFR | 1.5 | 202532_s_at | Dihydrofolate Reductase | 0.04 |
| TNRC9 | 1.5 | 215108_x_at | Trinucleotide Repeat Containing 9 | 0.02 |
| ***Lipid metabolism*** | |  |  |  |
| PCYT1A | -1.4 | 204209_at | Phosphate Cytidylyltransferase 1, Choline, Alpha | 0.002 |
| FDPS | 1.6 | 201275_at | Farnesyl Diphosphate Synthase (Farnesyl | 0.006 |
|  |  |  | Pyrophosphate Synthetase, Dimethylallyl |  |
|  |  |  | Transtransferase, Geranyltranstransferase) |  |
| PAFAH2 | 2 | 205232_s_at | Platelet-Activating Factor Acetyl- | 0.01 |
|  |  |  | Hydrolase 2, 40kDa |  |
| FADS1 | 1.7 | 208964_s_at | Fatty Acid Desaturase 1 | 9.5E-05 |
| SQLE | 2.5 | 213577_at | Squalene Epoxidase | 0.002 |
| SLC27A5 | 2 | 219733_s_at | Solute Carrier Family 27 (Fatty Acid | 0.02 |
|  |  |  | Transporter), Member 5 |  |
| EBP | 1.7 | 202735_at | Emopamil Binding Protein (Sterol Isomerase) | 0.03 |
|  |  |  |  |  |
| **Gene Symbol** | **Fold Change** | **Gene ID** | **Description** | **p Val** |
| HSD17B7 | 1.7 | 220081_x_at | Hydroxysteroid (17-Beta) Dehydrogenase 7 | 0.04 |
| ***Electron transport*** | |  |  |  |
| GCDH | -1.6 | 203500_at | Glutaryl-Coenzyme A Dehydrogenase | 0.02 |
| DHRS4 | 1.4 | 218021_at | Dehydrogenase/Reductase (SDR Family) | 0.01 |
|  |  |  | Member 4 |  |
| ***Others*** |  |  |  |  |
| YTHDF3 | -2.1 | 1564053_a_at | YTH Domain Family, Member 3 | 0.04 |
| GOLGA8B | -1.8 | 213650_at | Golgi Autoantigen, Golgin Subfamily A, 8B | 0.02 |
| TMEM63A | -1.5 | 202700_s_at | Transmembrane Protein 63A | 0.01 |
| MGC14376 | -1.7 | 214696_at | Hypothetical Protein MGC14376 | 0.03 |
| XTP3TPA | -1.7 | 218069_at | XTP3-Transactivated Protein A | 0.04 |
| ANKRD25 | -2 | 218418_s_at | Ankyrin Repeat Domain 25 | 0.04 |
| C12ORF49 | -2.3 | 218867_s_at | Chromosome 12 Open Reading Frame 49 | 0.03 |
| C14ORF103 | -1.4 | 219164_s_at | Chromosome 14 Open Reading Frame 103 | 0.02 |
| FLJ21820 | -1.9 | 222193_at | Hypothetical Protein Flj21820 | 0.02 |
| TMEM50B | -1.6 | 228001_at | Transmembrane Protein 50B | 0.04 |
| ZCCHC2 | -1.8 | 233425_at | Zinc Finger, CCHC Domain Containing 2 | 0.006 |
| C8ORF38 | -1.8 | 236766_at | Chromosome 8 Open Reading Frame 38 | 0.03 |
| FLJ21657 | -1.7 | 238635_at | Hypothetical Protein Flj21657 | 0.04 |
| FAM13C1 | 1.9 | 1554547_at | Family With Sequence Similarity13,Member C1 | 0.01 |
| LOC51336 | 2.2 | 208154_at | Mesenchymal Stem Cell Protein DSCD28 | 0.04 |
| TXNDC9 | 2.1 | 1554047_at | Thioredoxin Domain Containing 9 | 0.004 |
| CEP135 | 1.5 | 206003_at | Centrosomal Protein 135kDa | 0.02 |
| LOC171220 | 1.5 | 211325_x_at | Destrin-2 Pseudogene | 0.003 |
| KIAA0913 | 1.6 | 212359_s_at | KIAA0913 | 0.02 |
| HCG2P7 | 1.5 | 216229_x_at | HLA Complex Group 2 Pseudogene 7 | 0.04 |
| MALAT1 | 1.5 | 224567_x_at | Metastasis Associated Lung Adenocarcinoma | 0.02 |
|  |  |  | Transcript 1 (Non-Coding RNA) |  |
| C12ORF23 | 1.6 | 224759_s_at | Chromosome 12 Open Reading Frame 23 | 0.02 |
| KDELC2 | 1.7 | 225128_at | KDEL (Lys-Asp-Glu-Leu) Containing 2 | 0.005 |
| HCG12 | 1.5 | 227980_at | HLA Complex Group 12 | 0.03 |
| C10ORF58 | 1.5 | 228155_at | Chromosome 10 Open Reading Frame 58 | 0.04 |
| PHACTR4 | 3.3 | 233319_x_at | Phosphatase And Actin Regulator 4 | 0.03 |
| PGBD2 | 1.7 | 238004_at | PIGGYBAC Transposable Element Derived 2 | 0.047 |
| C5ORF3 | -1.5 | 218588_s_at | Chromosome 5 Open Reading Frame 3 | 0.046 |
| LYSMD4 | -1.7 | 228954_at | LYSM, Putative Peptidoglycan-Binding, | 0.01 |
|  |  |  | Domain Containing 4 |  |
| HHLA3 | -2 | 234665_x_at | HERV-H LTR-Associating 3 | 0.03 |
| MYOHD1 | -1.6 | 236022_at | Myosin Head Domain Containing 1 | 0.047 |
| LRRC8E | -1.42 | 239433_at | Leucine Rich Repeat Containing8 family MemberE | 0.007 |
| ZDHHC21 | -2 | 243835_at | Zinc Finger, DHHC-Type Containing 21 | 0.046 |
| NA | 1.7 | 214862_x_at | NA | 0.03 |
| NA | 1.5 | 229861_at | NA | 0.007 |
| NA | 1.8 | 235229_at | NA | 0.03 |
| NA | 1.6 | 236884_at | NA | 0.02 |
| NA | 2.3 | 241864_x_at | NA | 0.03 |
| NA | -1.6 | 1557149_at | NA | 0.03 |
| NA | -1.8 | 238012_at | NA | 0.01 |
|  |  |  |  |  |
