# Supplementary material for: Gene Expression Profiling and Network Analysis Reveals Lipid and Steroid Metabolism to Be the Most Favored by TNFα in HepG2 Cells
Source: PLoS One. 2010 Feb 4;5(2):e9063. doi: 10.1371/journal.pone.0009063 (PMC2816217; doi:10.1371/journal.pone.0009063)
Supplement: Table S2 — Gene Ontology (Biological Processes) terms overrepresented (p<0.01) in the set of genes altered by TNFα. (0.23 MB DOC) [file pone.0009063.s004.doc]

**Table S2: Gene Ontology (Biological Processes) terms Overrepresented (p<0.01) in the set of genes altered by TNFα.**

| **A. Overrepresentation of GO terms (Biological processes) among up regulated genes** | | | | |
| --- | --- | --- | --- | --- |
| **GO_ID** | **TERM** | **NB_IN_REF** | **NB_IN_SET** | **P_VALUE** |
| GO:0006694 | steroid biosynthetic process | 91 | 4 | 1.24E-05 |
| GO:0016126 | sterol biosynthetic process | 33 | 3 | 1.89E-05 |
| GO:0008202 | steroid metabolic process | 221 | 4 | 0.000374 |
| GO:0016125 | sterol metabolic process | 93 | 3 | 0.000412 |
| GO:0006695 | cholesterol biosynthetic process | 25 | 2 | 0.000701 |
| GO:0019752 | carboxylic acid metabolic process | 765 | 6 | 0.000998 |
| GO:0006082 | organic acid metabolic process | 767 | 6 | 0.001011 |
| GO:0046642 | negative regulation of alpha-beta T cell proliferation | 1 | 1 | 0.001576 |
| GO:0015942 | formate metabolic process | 1 | 1 | 0.001576 |
| GO:0008610 | lipid biosynthetic process | 332 | 4 | 0.001639 |
| GO:0006888 | ER to Golgi vesicle-mediated transport | 42 | 2 | 0.001962 |
| GO:0006629 | lipid metabolic process | 945 | 6 | 0.002775 |
| GO:0006097 | glyoxylate cycle | 2 | 1 | 0.003147 |
| GO:0002268 | follicular dendritic cell differentiation | 2 | 1 | 0.003147 |
| GO:0002266 | follicular dendritic cell activation | 2 | 1 | 0.003147 |
| GO:0051384 | response to glucocorticoid stimulus | 2 | 1 | 0.003147 |
| GO:0046636 | negative regulation of alpha-beta T cell activation | 2 | 1 | 0.003147 |
| GO:0045333 | cellular respiration | 59 | 2 | 0.003801 |
| GO:0046640 | regulation of alpha-beta T cell proliferation | 3 | 1 | 0.004713 |
| GO:0046487 | glyoxylate metabolic process | 3 | 1 | 0.004713 |
| GO:0006545 | glycine biosynthetic process | 3 | 1 | 0.004713 |
| GO:0044255 | cellular lipid metabolic process | 755 | 5 | 0.005269 |
| GO:0046633 | alpha-beta T cell proliferation | 4 | 1 | 0.006274 |
| GO:0046634 | regulation of alpha-beta T cell activation | 4 | 1 | 0.006274 |
| GO:0008652 | amino acid biosynthetic process | 77 | 2 | 0.006327 |
| GO:0008203 | cholesterol metabolic process | 77 | 2 | 0.006327 |
| GO:0043062 | extracellular structure organization and biogenesis | 84 | 2 | 0.007459 |
| GO:0002467 | germinal center formation | 5 | 1 | 0.007831 |
| GO:0048536 | spleen development | 5 | 1 | 0.007831 |
| GO:0048193 | Golgi vesicle transport | 91 | 2 | 0.00867 |
| GO:0032787 | monocarboxylic acid metabolic process | 289 | 3 | 0.009474 |
| GO:0006886 | intracellular protein transport | 565 | 4 | 0.009902 |
|  |  |  |  |  |
| **B. Overrepresentation of GO terms (Biological processes) among down regulated genes** | | | | |
| **GO_ID** | **TERM** | **NB_IN_REF** | **NB_IN_SET** | **P_VALUE** |
| GO:0031497 | chromatin assembly | 194 | 6 | 1.48E-06 |
| GO:0022607 | cellular component assembly | 809 | 10 | 1.65E-06 |
| GO:0006333 | chromatin assembly or disassembly | 257 | 6 | 7.37E-06 |
| GO:0065003 | macromolecular complex assembly | 755 | 9 | 7.74E-06 |
| GO:0002526 | acute inflammatory response | 78 | 4 | 1.31E-05 |
| GO:0006325 | establishment and/or maintenance of chromatin | 438 | 7 | 1.40E-05 |
|  | architecture |  |  |  |
| GO:0006323 | DNA packaging | 444 | 7 | 1.52E-05 |
|  |  |  |  |  |
|  |  |  |  |  |
|  |  |  |  |  |
| **GO_ID** | **TERM** | **NB_IN_REF** | **NB_IN_SET** | **P_VALUE** |
| GO:0006334 | nucleosome assembly | 176 | 5 | 1.77E-05 |
| GO:0007001 | chromosome organization and biogenesis (sensu | 523 | 7 | 4.25E-05 |
|  | Eukaryota) |  |  |  |
| GO:0019884 | antigen processing and presentation of | 6 | 2 | 5.03E-05 |
|  | exogenous antigen |  |  |  |
| GO:0051276 | chromosome organization and biogenesis | 543 | 7 | 5.36E-05 |
| GO:0043170 | macromolecule metabolic process | 10891 | 33 | 9.19E-05 |
| GO:0016043 | cellular component organization and biogenesis | 3255 | 16 | 0.000114 |
| GO:0065004 | protein-DNA complex assembly | 269 | 5 | 0.000129 |
| GO:0016064 | immunoglobulin mediated immune response | 54 | 3 | 0.000136 |
| GO:0019724 | B cell mediated immunity | 55 | 3 | 0.000144 |
| GO:0006950 | response to stress | 1182 | 9 | 0.000229 |
| GO:0044238 | primary metabolic process | 12554 | 35 | 0.000264 |
| GO:0045184 | establishment of protein localization | 949 | 8 | 0.000271 |
| GO:0002253 | activation of immune response | 71 | 3 | 0.000305 |
| GO:0002449 | lymphocyte mediated immunity | 73 | 3 | 0.000331 |
| GO:0008104 | protein localization | 991 | 8 | 0.000359 |
| GO:0002443 | leukocyte mediated immunity | 78 | 3 | 0.000401 |
| GO:0002250 | adaptive immune response | 82 | 3 | 0.000464 |
| GO:0002460 | adaptive immune response based on somatic | 82 | 3 | 0.000464 |
|  | recombination of immune receptors built from |  |  |  |
|  | immunoglobulin superfamily domains |  |  |  |
| GO:0002684 | positive regulation of immune system process | 83 | 3 | 0.00048 |
| GO:0050778 | positive regulation of immune response | 83 | 3 | 0.00048 |
| GO:0033036 | macromolecule localization | 1042 | 8 | 0.000495 |
| GO:0006952 | defense response | 570 | 6 | 0.000541 |
| GO:0043066 | negative regulation of apoptosis | 219 | 4 | 0.000675 |
| GO:0008152 | metabolic process | 14272 | 37 | 0.00068 |
| GO:0042742 | defense response to bacterium | 95 | 3 | 0.000709 |
| GO:0043069 | negative regulation of programmed cell death | 222 | 4 | 0.000709 |
| GO:0050776 | regulation of immune response | 96 | 3 | 0.00073 |
| GO:0002682 | regulation of immune system process | 96 | 3 | 0.00073 |
| GO:0009611 | response to wounding | 409 | 5 | 0.000839 |
| GO:0051240 | positive regulation of multicellular organismal | 101 | 3 | 0.000844 |
|  | process |  |  |  |
| GO:0050789 | regulation of biological process | 6041 | 21 | 0.000867 |
| GO:0002252 | immune effector process | 102 | 3 | 0.000868 |
| GO:0065007 | biological regulation | 6566 | 22 | 0.001005 |
| GO:0009617 | response to bacterium | 108 | 3 | 0.001022 |
| GO:0015031 | protein transport | 900 | 7 | 0.001048 |
| GO:0006958 | complement activation, classical pathway | 28 | 2 | 0.00122 |
| GO:0065008 | regulation of biological quality | 679 | 6 | 0.001296 |
| GO:0002455 | humoral immune response mediated by | 30 | 2 | 0.001399 |
|  | circulating immunoglobulin |  |  |  |
| GO:0006259 | DNA metabolic process | 1247 | 8 | 0.001504 |
| GO:0009607 | response to biotic stimulus | 279 | 4 | 0.001613 |
|  |  |  |  |  |
|  |  |  |  |  |
|  |  |  |  |  |
| **GO_ID** | **TERM** | **NB_IN_REF** | **NB_IN_SET** | **P_VALUE** |
| GO:0006954 | inflammatory response | 283 | 4 | 0.001696 |
| GO:0001812 | positive regulation of type I hypersensitivity | 1 | 1 | 0.001859 |
| GO:0001802 | type III hypersensitivity | 1 | 1 | 0.001859 |
|  |  |  |  |  |
| GO:0051136 | regulation of NK T cell differentiation | 1 | 1 | 0.001859 |
| GO:0001810 | regulation of type I hypersensitivity | 1 | 1 | 0.001859 |
| GO:0033028 | myeloid cell apoptosis | 1 | 1 | 0.001859 |
| GO:0048007 | antigen processing and presentation, exogenous | 1 | 1 | 0.001859 |
|  | lipid antigen via MHC class Ib |  |  |  |
| GO:0051043 | regulation of membrane protein ectodomain | 1 | 1 | 0.001859 |
|  | proteolysis |  |  |  |
| GO:0033025 | regulation of mast cell apoptosis | 1 | 1 | 0.001859 |
| GO:0033024 | mast cell apoptosis | 1 | 1 | 0.001859 |
| GO:0002431 | Fc receptor mediated stimulatory signaling pathway | 1 | 1 | 0.001859 |
| GO:0001865 | NK T cell differentiation | 1 | 1 | 0.001859 |
| GO:0033023 | mast cell homeostasis | 1 | 1 | 0.001859 |
| GO:0033032 | regulation of myeloid cell apoptosis | 1 | 1 | 0.001859 |
| GO:0001803 | regulation of type III hypersensitivity | 1 | 1 | 0.001859 |
| GO:0051045 | negative regulation of membrane protein | 1 | 1 | 0.001859 |
|  | ectodomain proteolysis |  |  |  |
| GO:0033033 | negative regulation of myeloid cell apoptosis | 1 | 1 | 0.001859 |
| GO:0033026 | negative regulation of mast cell apoptosis | 1 | 1 | 0.001859 |
| GO:0042590 | antigen processing and presentation of exogenous | 1 | 1 | 0.001859 |
|  | peptide antigen via MHC class I |  |  |  |
| GO:0022617 | extracellular matrix disassembly | 1 | 1 | 0.001859 |
| GO:0032733 | positive regulation of interleukin-10 production | 1 | 1 | 0.001859 |
| GO:0051138 | positive regulation of NK T cell differentiation | 1 | 1 | 0.001859 |
| GO:0001805 | positive regulation of type III hypersensitivity | 1 | 1 | 0.001859 |
| GO:0031581 | hemidesmosome assembly | 1 | 1 | 0.001859 |
| GO:0006956 | complement activation | 40 | 2 | 0.002463 |
| GO:0002541 | activation of plasma proteins during acute | 40 | 2 | 0.002463 |
|  | inflammatory response |  |  |  |
| GO:0051246 | regulation of protein metabolic process | 315 | 4 | 0.002469 |
| GO:0042981 | regulation of apoptosis | 550 | 5 | 0.002926 |
| GO:0043067 | regulation of programmed cell death | 556 | 5 | 0.003059 |
| GO:0048523 | negative regulation of cellular process | 1106 | 7 | 0.003175 |
| GO:0006915 | apoptosis | 826 | 6 | 0.0033 |
| GO:0012501 | programmed cell death | 833 | 6 | 0.003431 |
| GO:0006916 | anti-apoptosis | 170 | 3 | 0.003612 |
| GO:0002866 | positive regulation of acute inflammatory response | 2 | 1 | 0.00371 |
|  | to antigenic stimulus |  |  |  |
| GO:0045921 | positive regulation of exocytosis | 2 | 1 | 0.00371 |
| GO:0002886 | regulation of myeloid leukocyte mediated immunity | 2 | 1 | 0.00371 |
| GO:0002712 | regulation of B cell mediated immunity | 2 | 1 | 0.00371 |
| GO:0002675 | positive regulation of acute inflammatory response | 2 | 1 | 0.00371 |
| GO:0032755 | positive regulation of interleukin-6 production | 2 | 1 | 0.00371 |
| GO:0002885 | positive regulation of hypersensitivity | 2 | 1 | 0.00371 |
| GO:0001796 | regulation of type IIa hypersensitivity | 2 | 1 | 0.00371 |
|  |  |  |  |  |
|  |  |  |  |  |
| **GO_ID** | **TERM** | **NB_IN_REF** | **NB_IN_SET** | **P_VALUE** |
| GO:0001794 | type IIa hypersensitivity | 2 | 1 | 0.00371 |
| GO:0043304 | regulation of mast cell degranulation | 2 | 1 | 0.00371 |
| GO:0002888 | positive regulation of myeloid leukocyte mediated | 2 | 1 | 0.00371 |
|  | immunity |  |  |  |
| GO:0002891 | positive regulation of immunoglobulin mediated | 2 | 1 | 0.00371 |
|  | immune response |  |  |  |
| GO:0043306 | positive regulation of mast cell degranulation | 2 | 1 | 0.00371 |
| GO:0002714 | positive regulation of B cell mediated immunity | 2 | 1 | 0.00371 |
| GO:0001798 | positive regulation of type IIa hypersensitivity | 2 | 1 | 0.00371 |
| GO:0048003 | antigen processing and presentation of lipid | 2 | 1 | 0.00371 |
|  | antigen via MHC class Ib |  |  |  |
| GO:0002445 | type II hypersensitivity | 2 | 1 | 0.00371 |
| GO:0032653 | regulation of interleukin-10 production | 2 | 1 | 0.00371 |
| GO:0043302 | positive regulation of leukocyte degranulation | 2 | 1 | 0.00371 |
| GO:0002863 | positive regulation of inflammatory response to | 2 | 1 | 0.00371 |
|  | antigenic stimulus |  |  |  |
| GO:0032675 | regulation of interleukin-6 production | 2 | 1 | 0.00371 |
| GO:0032765 | positive regulation of mast cell cytokine production | 2 | 1 | 0.00371 |
| GO:0002894 | positive regulation of type II hypersensitivity | 2 | 1 | 0.00371 |
| GO:0002889 | regulation of immunoglobulin mediated immune | 2 | 1 | 0.00371 |
|  | response |  |  |  |
| GO:0002892 | regulation of type II hypersensitivity | 2 | 1 | 0.00371 |
| GO:0002475 | antigen processing and presentation via MHC classIb | 2 | 1 | 0.00371 |
| GO:0006461 | protein complex assembly | 355 | 4 | 0.003726 |
| GO:0009605 | response to external stimulus | 587 | 5 | 0.00381 |
| GO:0048519 | negative regulation of biological process | 1153 | 7 | 0.003935 |
| GO:0006996 | organelle organization and biogenesis | 1476 | 8 | 0.004006 |
| GO:0016265 | death | 878 | 6 | 0.004367 |
| GO:0008219 | cell death | 878 | 6 | 0.004367 |
| GO:0006417 | regulation of translation | 185 | 3 | 0.00454 |
| GO:0040029 | regulation of gene expression, epigenetic | 57 | 2 | 0.00489 |
| GO:0010324 | membrane invagination | 194 | 3 | 0.005158 |
| GO:0006897 | endocytosis | 194 | 3 | 0.005158 |
| GO:0048468 | cell development | 1223 | 7 | 0.005299 |
| GO:0032763 | regulation of mast cell cytokine production | 3 | 1 | 0.005556 |
| GO:0002861 | regulation of inflammatory response to antigenic | 3 | 1 | 0.005556 |
|  | stimulus |  |  |  |
| GO:0016068 | type I hypersensitivity | 3 | 1 | 0.005556 |
| GO:0002864 | regulation of acute inflammatory response to | 3 | 1 | 0.005556 |
|  | antigenic stimulus |  |  |  |
| GO:0043300 | regulation of leukocyte degranulation | 3 | 1 | 0.005556 |
| GO:0032760 | positive regulation of tumor necrosis factor production | 3 | 1 | 0.005556 |
| GO:0002673 | regulation of acute inflammatory response | 3 | 1 | 0.005556 |
| GO:0032762 | mast cell cytokine production | 3 | 1 | 0.005556 |
| GO:0002883 | regulation of hypersensitivity | 3 | 1 | 0.005556 |
|  |  |  |  |  |
|  |  |  |  |  |
| **GO_ID** | **TERM** | **NB_IN_REF** | **NB_IN_SET** | **P_VALUE** |
| GO:0051707 | response to other organism | 200 | 3 | 0.005595 |
| GO:0031326 | regulation of cellular biosynthetic process | 202 | 3 | 0.005745 |
| GO:0009889 | regulation of biosynthetic process | 217 | 3 | 0.006944 |
| GO:0007044 | cell-substrate junction assembly | 4 | 1 | 0.007394 |
| GO:0002478 | antigen processing and presentation of exogenous | 4 | 1 | 0.007394 |
|  | peptide antigen |  |  |  |
| GO:0042091 | interleukin-10 biosynthetic process | 4 | 1 | 0.007394 |
| GO:0045074 | regulation of interleukin-10 biosynthetic process | 4 | 1 | 0.007394 |
| GO:0006622 | protein targeting to lysosome | 4 | 1 | 0.007394 |
| GO:0045082 | positive regulation of interleukin-10 biosynthetic | 4 | 1 | 0.007394 |
|  | process |  |  |  |
| GO:0002495 | antigen processing and presentation of peptide | 4 | 1 | 0.007394 |
|  | antigen via MHC class II |  |  |  |
| GO:0002262 | myeloid cell homeostasis | 4 | 1 | 0.007394 |
| GO:0032680 | regulation of tumor necrosis factor production | 4 | 1 | 0.007394 |
| GO:0019886 | antigen processing and presentation of exogenous | 4 | 1 | 0.007394 |
|  | peptide antigen via MHC class II |  |  |  |
| GO:0006605 | protein targeting | 223 | 3 | 0.00746 |
| GO:0044237 | cellular metabolic process | 12448 | 31 | 0.007953 |
| GO:0042592 | homeostatic process | 451 | 4 | 0.008253 |
| GO:0006959 | humoral immune response | 76 | 2 | 0.008441 |
| GO:0016477 | cell migration | 235 | 3 | 0.008554 |
| GO:0048518 | positive regulation of biological process | 1029 | 6 | 0.0088 |
| GO:0050896 | response to stimulus | 3272 | 12 | 0.008896 |
| GO:0043303 | mast cell degranulation | 5 | 1 | 0.009226 |
| GO:0006911 | phagocytosis, engulfment | 5 | 1 | 0.009226 |
| GO:0002448 | mast cell mediated immunity | 5 | 1 | 0.009226 |
| GO:0045861 | negative regulation of proteolysis | 5 | 1 | 0.009226 |
| GO:0006656 | phosphatidylcholine biosynthetic process | 5 | 1 | 0.009226 |
| GO:0043249 | erythrocyte maturation | 5 | 1 | 0.009226 |
| GO:0032613 | interleukin-10 production | 5 | 1 | 0.009226 |
| GO:0002437 | inflammatory response to antigenic stimulus | 5 | 1 | 0.009226 |
| GO:0002438 | acute inflammatory response to antigenic stimulus | 5 | 1 | 0.009226 |
| GO:0046470 | phosphatidylcholine metabolic process | 5 | 1 | 0.009226 |
| GO:0002524 | hypersensitivity | 5 | 1 | 0.009226 |
|  |  |  |  |  |
|  |  |  |  |  |
|  |  |  |  |  |
|  |  |  |  |  |
|  |  |  |  |  |
|  |  |  |  |  |
|  |  |  |  |  |
|  |  |  |  |  |
|  |  |  |  |  |
